# Mapping local cluster separation, global community structure, and leadership concentration among the 2025 top 2% dermatology scientists: Beyond rankings

**DOI:** 10.1097/MD.0000000000050851

**Published:** 2026-10-09

**Authors:** Yu-An Wang, Po-Chih Lai, Feng-Jie Lai

**Affiliations:** a Department of Dermatology, Chi Mei Medical Center, Tainan, Taiwan; b School of Medicine, National Yang Ming Chiao Tung University, Hsinchu, Taiwan.

**Keywords:** absolute advantage coefficient, dermatology network visualization, modularity Q*, silhouette plot, top 2% scientists

## Abstract

**Background:**

The Stanford/Ioannidis top 2% scientists database provides standardized citation-based indicators for identifying influential researchers, but conventional rankings offer limited information about the structural organization of research communities and collaboration patterns. This study aimed to develop and demonstrate an integrated bibliometric reporting framework for evaluating local cluster separation, global community structure, and leadership concentration among the 2025 top 2% dermatology scientists.

**Methods:**

Data for 463 dermatology scientists from 35 countries and 360 institutions were analyzed. Country-, institution-, and coauthor-based networks were constructed and evaluated using 3 complementary metrics: silhouette scores (SS) for local cluster separation, normalized modularity (Q*) for global community structure, and the absolute advantage coefficient (AAC) for leadership concentration. Network maps, silhouette plots, profile summaries, and Kano-style visualizations were used to facilitate interpretation. Dirk Schadendorf and Michael R. Hamblin were selected as illustrative scholar-level cases based on their citation and field-ranking prominence.

**Results:**

The top 100 high-frequency country and institutional nodes formed 15 clusters. The United States, Germany, the United Kingdom, Australia, and Sweden were the most prominent countries, with Harvard Medical School, Charité–Universitätsmedizin Berlin, and the University of California, San Francisco emerging as major institutional hubs. Schadendorf was the most highly cited scholar, whereas Hamblin ranked highest within dermatology. Their coauthor networks showed similar local and global cluster characteristics but different leadership-concentration patterns: Schadendorf had SS = 0.70, Q* = 0.75, and AAC = 0.66, whereas Hamblin had SS = 0.71, Q* = 0.74, and AAC = 0.76. Under the proposed AAC classification, these values indicate more distributed multicore collaboration for Schadendorf and greater single-core leadership concentration for Hamblin.

**Conclusion:**

Integrating SS, Q*, and AAC provides complementary information on local cluster separation, global community organization, and leadership concentration that cannot be obtained from citation rankings alone. The framework offers a reproducible and interpretable approach for research mapping, comparative assessment, and longitudinal monitoring across scientific disciplines. AAC should be interpreted as an indicator of collaboration leadership concentration rather than direct evidence of mentorship.

**Highlights**

1. Integrates SS, Q*, and AAC to assess 3 complementary dimensions of bibliometric networks.
2. Maps 463 top 2% dermatology scientists across 35 countries and 360 institutions.
3. Distinguishes local cluster separation, global community structure, and leadership concentration.
4. Identifies major countries, institutional hubs, and representative scholars through interpretable visualizations.
5. Demonstrates that citation prominence and collaboration leadership concentration represent distinct dimensions of scholarly performance.

## 1. Introduction

Since 2019, Ioannidis and collaborators have established the Standardized Citation Indicators Database for All Scientists (hosted by Elsevier/Mendeley Data), which is updated annually.^[1–3]^ This database, derived from Scopus, covers the entire citation record of researchers’ academic careers and classifies them into 22 disciplines and 176 subfields, providing standardized indicators for cross-disciplinary comparison.^[1–5]^

Based on these data, Ioannidis team calculates multiple metrics (including total citations, *h*-index, and a composite *c*-score) focusing on a “citation impact score” to label and rank over 200,000 scientists.^[1,6]^ However, career-long citation–based rankings tend to favor senior scholars. Current media coverage also focuses mainly on name lists and headcounts, rarely combining interpretable visualizations and quantitative indicators to evaluate or summarize individual scholarly portfolios.

### 1.1. Weighted contributions of first and corresponding authors

Although the *h*-index is widely used, it favors senior researchers^[6]^ and includes all coauthors in its calculation. Even though Ioannidis and colleagues have removed self-citations and applied cross-field normalization to develop the *c*-score,^[1,7–9]^ their most recent release (August 2024)^[10]^ still disadvantages early-career researchers,^[11]^ as all coauthors remain included in ranking statistics.

Beyond the ranking of top 2% scientists,^[1–3]^ it is important to consider first- and corresponding-author contributions and to present these patterns through interpretable visualizations.^[12–15]^ In particular, collaboration structures may help identify whether scholarly output is concentrated around a leading investigator or distributed across several core contributors. For Top 2% scientists,^[10]^ such patterns are relevant to assessing academic leadership and the potential capacity to guide junior researchers within collaborative teams.

However, network-based indicators describe collaboration concentration rather than direct mentor–mentee relationships. This methodological gap calls for an integrated framework that transparently evaluates cluster structure, collaboration patterns, and leadership concentration.

### 1.2. Visualization of textual and relational data

Methodologically, studies using the top 2% scientists dataset often visualize numeric data (e.g., citation metrics or *c*-scores) with simple tables or bar charts.^[2,3,11]^ In contrast, textual and relational data (such as co-occurrences of authors, countries, institutions, and disciplines) are seldom weighted or analyzed using community-detection approaches.^[16–20]^

In other words, conventional network diagrams rarely include quantifiable measures of cluster quality, such as “within-cluster cohesion” and “between-cluster separation,” analogous to reporting means without variances or standard deviations.

Although bivariate scatterplots^[17]^ and network graphs^[21–28]^ can complement each other, few studies integrate them with silhouette scores (SS),^[20,29]^ absolute advantage coefficient (AAC),^[30–32]^ or normalized modularity Q* (= Qw/Q_max_).^[24,33]^ Commercial software packages (e.g., VOSviewer^[23]^ and CiteSpace^[34]^) also lack built-in support or demonstrations for SS, AAC, and Q*.

### 1.3. Study contribution and analytic framework

This study does not develop a new clustering algorithm or bibliometric indicator. Instead, it presents an integrated reporting framework that combines SS, Q*, and AAC to assess complementary features of bibliometric networks: local cluster separation, global community structure, and leadership concentration. By integrating these quantitative measures with network and profile visualizations, the framework makes the quality and interpretation of network maps more transparent. We demonstrate its application using the 2025 top 2% dermatology-scientist dataset.

### 1.4. Research objectives

This study aims to develop and demonstrate an integrated bibliometric reporting framework for the 2025 Top 2% dermatology-scientist dataset by focusing on 3 complementary dimensions of network structure: local cluster separation, assessed using SS^[20,29]^ to evaluate within-cluster cohesion and between-cluster distinction in co-word and institutional networks; global community structure, assessed using normalized modularity (Q*)^[24,31]^ to quantify the overall strength and organization of network communities; and leadership concentration, assessed using the AAC^[30–32]^ to characterize the extent to which scholarly contributions are concentrated around leading investigators versus distributed among multiple core contributors, with particular attention to its potential relevance for mentoring junior researchers.^[30]^ These measures are integrated with network and profile visualizations to improve the transparency, interpretability, and quantitative evaluation of bibliometric maps.

## 2. Methods

### 2.1. Data source

The 2025 Stanford/Ioannidis top 2% scientists dataset^[10]^ was downloaded, and scholars affiliated with their institutions were identified. A total of 463 scholars from 35 countries and 360 institutions were analyzed, except for 1 author (Samuel Ayres) whose country and institution were unspecified. Study data and instructions for drawing visualizations are deposited in Supplemental Digital Contents 1, 2, and 3.

This study did not involve human participants or medical records and was exempt from institutional ethics review.

### 2.2. Network-quality and leadership metrics

We used SS,^[20,29,35–37]^ Q*,^[33]^ and AAC^[30–32]^ because they assess complementary properties of the collaboration network.

SS measures local cluster quality by comparing within-cluster cohesion with separation from the nearest neighboring cluster. Q* measures global community structure, normalized for the number of detected communities, thereby enabling comparison across networks with different cluster counts. AAC measures the relative concentration of collaboration or output in the leading node compared with the next 2 nodes. Together, these metrics describe local separation (SS), global partitioning (Q*), and leadership concentration (AAC). Detailed formulas and worked examples are provided in Supplemental Digital Content 4.

To facilitate interpretation of AAC, Table 1^[38]^ presents a 5-level classification based on its corresponding odds, *r* = AAC/(1 − AAC). We used AAC = 0.70 (*r* = 2.33) as a practical, literature-based rule-of-thumb indicating substantial leadership concentration. This value distinguishes multicore from predominantly single-core collaboration patterns. The 0.70 value is not used as a universal quality cutoff for SS or Q*, and it should not be interpreted as direct evidence of mentoring.

**Table 1:** Five-level classification of AAC for mentoring junior researchers.

AAC	Odds *r*	Log-odds ln(r)	Five-level classification
AAC < 0.60	*R* < 1.5	< 0.405	Very weak mentoring (dual-core collaboration)
0.60 ≤ AAC < 0.67	1.5 ≤ *R* < 2	0.405–0.693	Weak mentoring (multicore collaboration)
0.67 ≤ AAC < 0.70	2 ≤ *R* < 2.33	0.693–0.847	Moderately strong mentoring (multicore collaboration)
Threshold: 0.70	*r* = 2.33	0.847	Practical rule-of-thumb
0.70 ≤ AAC < 0.75	2.33 ≤ *R* < 3	0.847–1.10	Very strong mentoring (single-core leadership)
AAC ≥ 0.75	*R* ≥ 3	≥1.10	Extremely strong mentoring (single-core leadership)

AAC = r/(1 + r), where r = AAC/(1 − AAC), and ln(r) denotes the log-odds.

AAC = absolute advantage coefficient.

### 2.3. Analysis and visualization

Cleaned co-word and coauthor data were analyzed in R using following follower clustering algorithm.^[16,30,39,40]^ We generated network, silhouette, and Kano-style visualizations and report the full analytic workflow, code, software versions, and plotting specifications in Supplemental Digital Contents 3 and 4, accompanying code repository in Github.^[36,37,41,42]^

### 2.4. Construction of the silhouette plot

The cleaned co-word dataset (e.g., country.csv) was processed using R scripts^[43]^ to generate silhouette plots of quantitative indicators, complementing traditional network and Kano diagrams.^[44]^ All data processing and statistical analyses were conducted using R software (version 4.1.3; R Foundation for Statistical Computing),^[45]^ and visualizations as well as output generation were performed within the RStudio integrated development environment.^[46]^ The approaches to conducting this study are deposited in Supplemental Digital Content 3.

## 3. Results

### 3.1. Country- and institution-based network

The analysis included 463 scholars from 35 countries and 360 institutions. The top 100 high-frequency nodes formed 15 clusters. Figure 1 presents the silhouette plot and force-directed network map of country- and institution-level co-occurrence patterns. The United States (US), Germany, the United Kingdom (UK), Australia, and Sweden were the most frequently represented countries. The network metrics reported in Figure 1 included SS, weighted modularity, unweighted modularity, AAC, and normalized modularity (Q* =1.00; k = 5, Q_max_ = 0.80).

**Figure 1.**
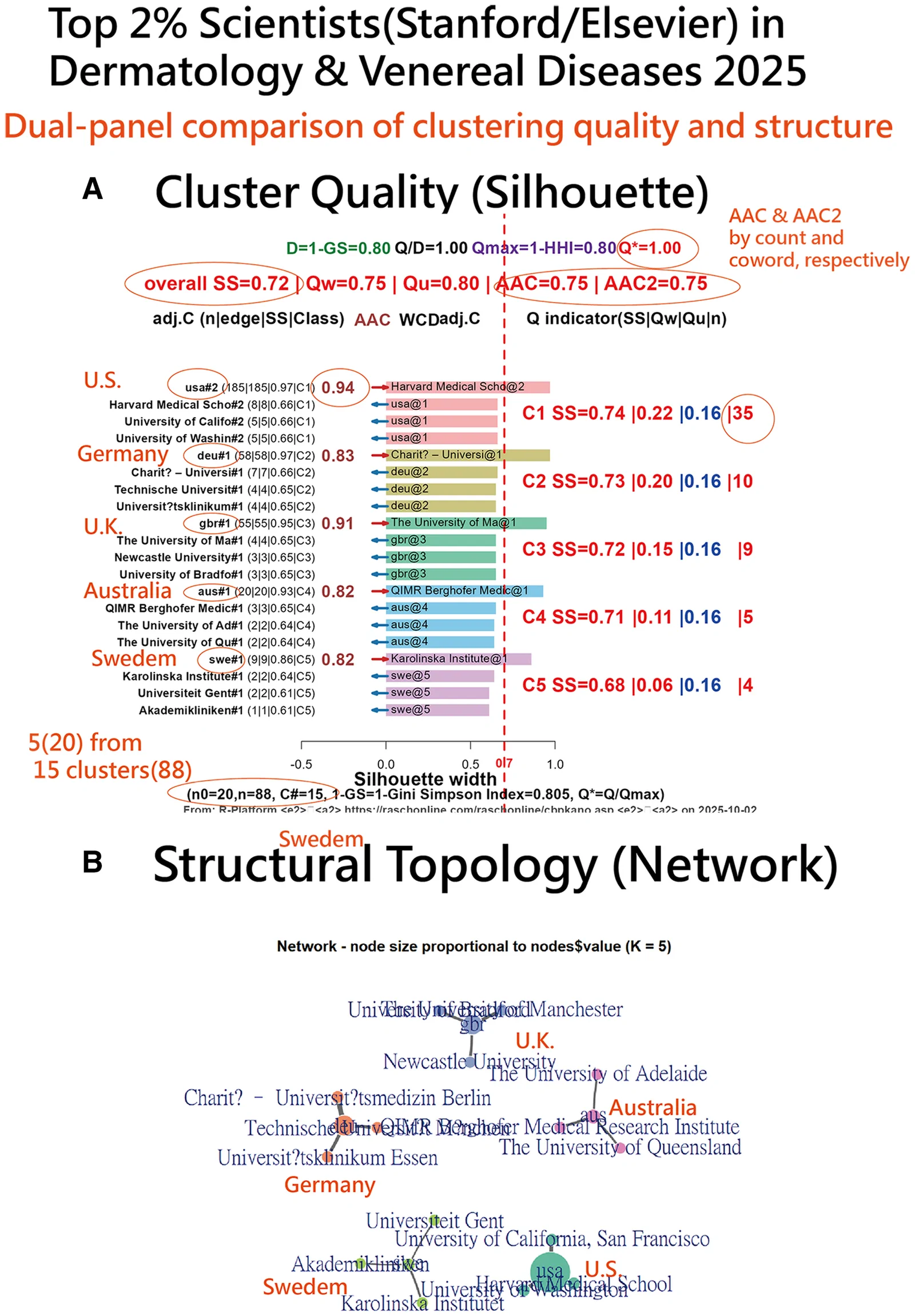
Dual-panel comparison of clustering quality and structure (left) silhouette panel summarizing within-cluster cohesion and between-cluster separation. (Right) Force-directed network visualization showing inter-node connectivity and community organization among the top 2% dermatology scientists. AAC = absolute advantage coefficient, SS = silhouette scores.

### 3.2. Distribution of leading dermatology scientists

Figure 2 displays publication and citation counts for scientists in the 2025 Stanford/Ioannidis top 2% list. Each point represents 1 scientist; point size corresponds to publication count and color corresponds to ranking. Dr Dirk Schadendorf (Germany) and Michael R. Hamblin (US) were selected for subsequent profile and collaboration network analyses because of their citation and publication/ranking positions in the plot.

**Figure 2.**
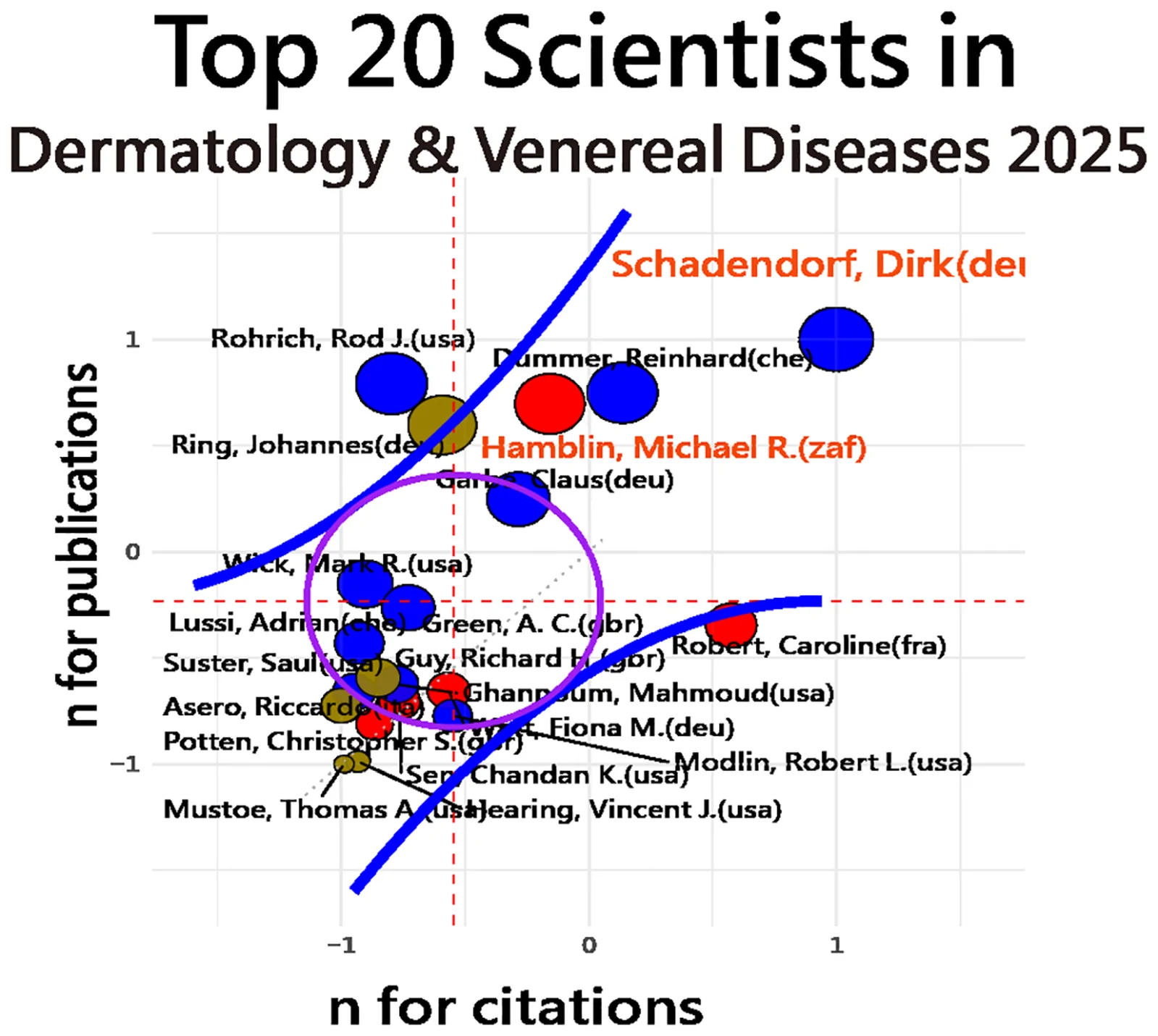
Top 20 scientists in Dermatology & Venereal Diseases (2025). Scatterplot illustrating leading dermatology scientists from the 2025 Stanford/Ioannidis top 2% list. Each point represents 1 scholar, with node size proportional to publication count and color indicating ranking).

### 3.3. Profile and collaboration network of Dirk Schadendorf

Figure 3 summarizes the bibliometric profile of Dr Dirk Schadendorf, including 983 publications and an *h*-index^[6]^ of 126. The profile displays distributions by country, institution, department, journal, and research domain. Germany, University Hospital Essen, and oncology-related research terms were the most frequent entries in the respective profile components. The overall AAC was 0.73.

**Figure 3.**
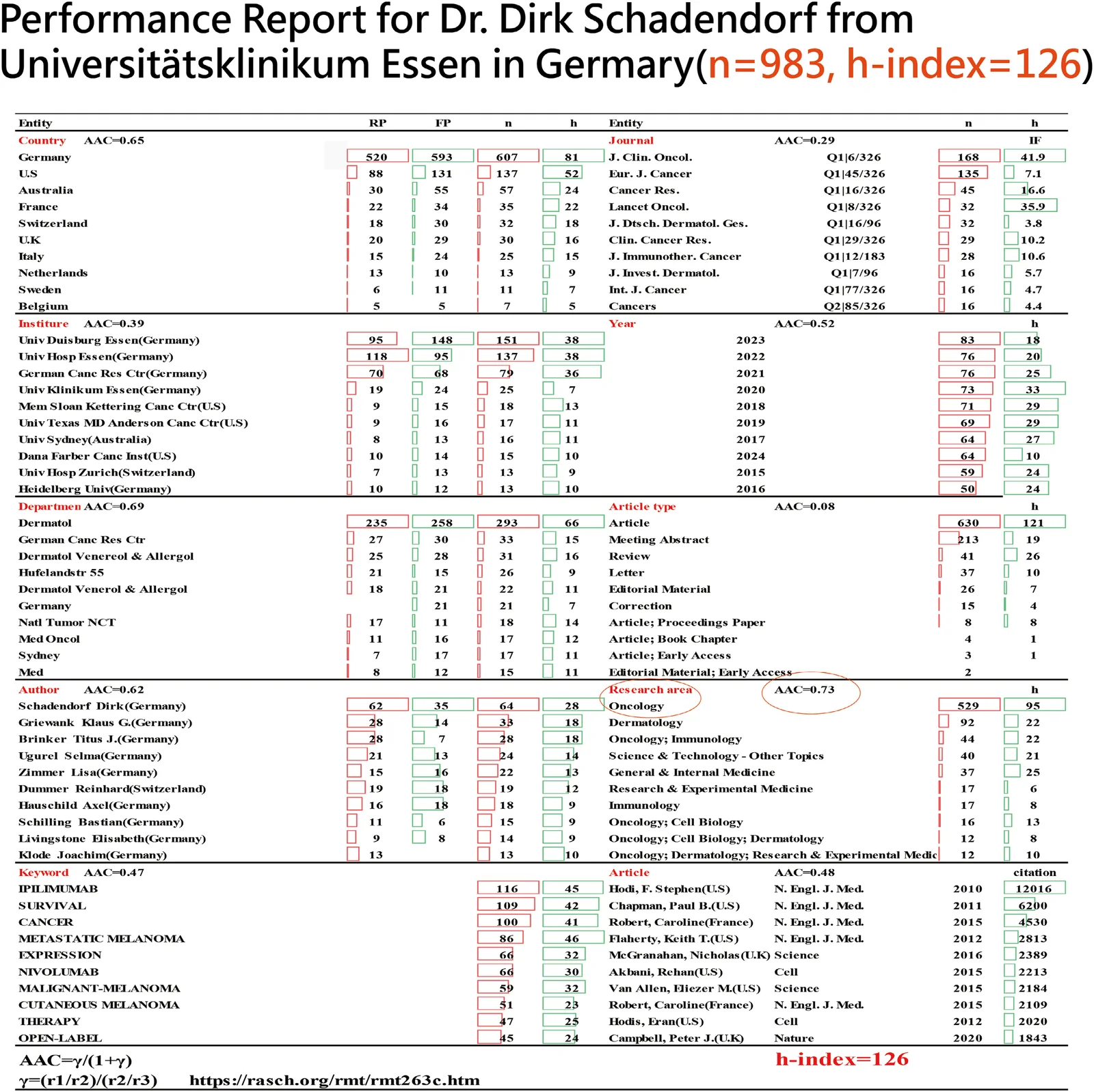
Performance report for Dr Dirk Schadendorf (global rank: 6774 among top 2% scientists). Comprehensive bibliometric profile summarizing publication and citation indicators (n = 983; h-index = 126), highlighting Germany, the University Hospital Essen, and oncology-related research as dominant areas. The AAC (0.73 > 0.70) identifies his leading influence in dermatology and oncology research networks. AAC = absolute advantage coefficient.

Figure 4 presents Dr Schadendorf coauthor network, comprising 47 nodes and 13 clusters. The overall SS was 0.70, Q* was 0.75, and AAC was 0.66. Lisa Zimmer and Titus Brinker were among the nodes with higher collaboration frequencies.

**Figure 4.**
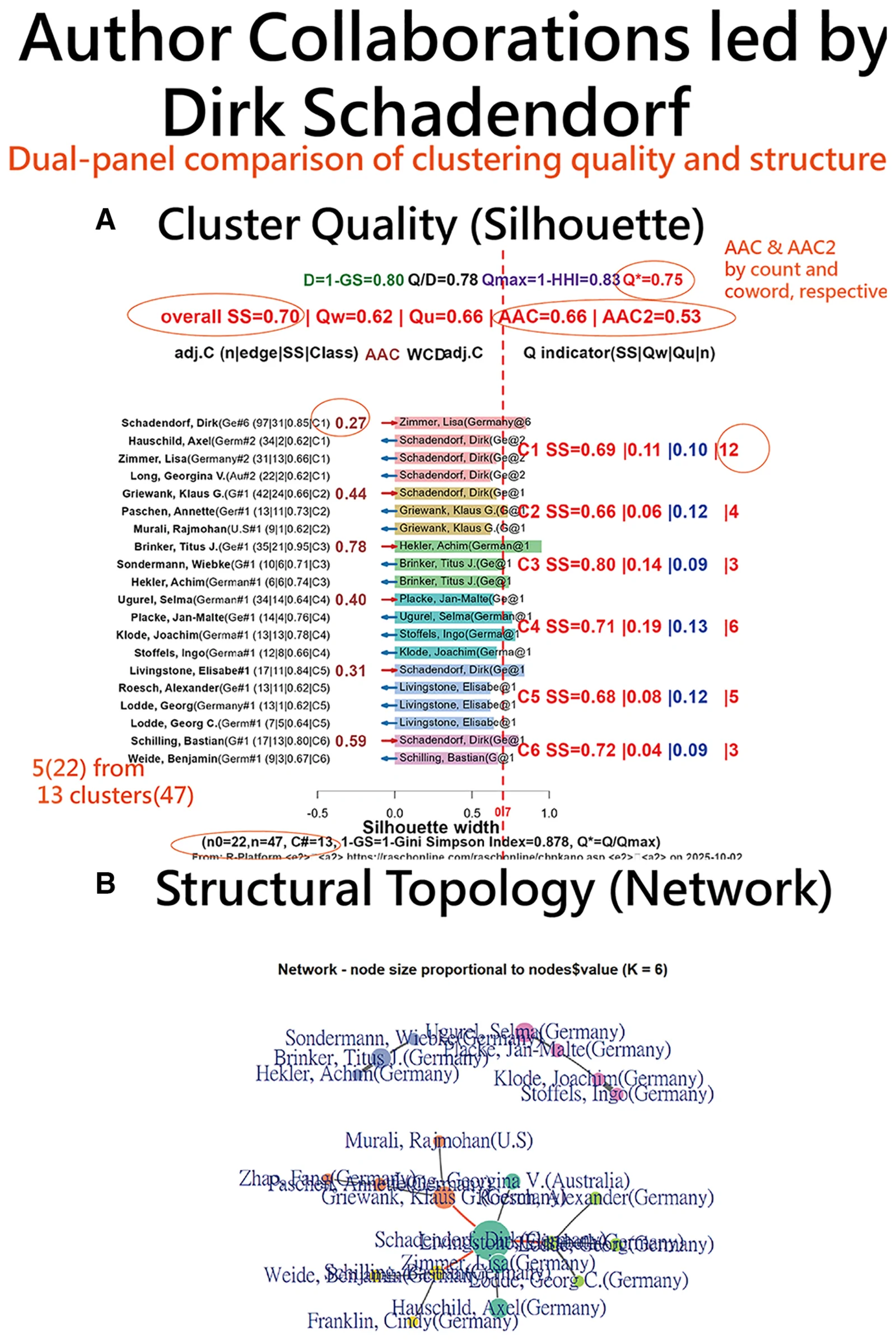
Silhouette plot of author collaborations led by Dr Dirk Schadendorf (rank 6774 among top 2% scientists). Dual-panel visualization of collaboration clusters (n = 47; k = 13). The silhouette panel (top) quantifies cluster cohesion and separation (overall SS = 0.70, Q* = 0.75, AAC = 0.66), while the network panel (bottom) maps coauthor relationships, highlighting key collaborators such as Lisa Zimmer and Titus Brinker. AAC = absolute advantage coefficient, SS = silhouette scores.

Figure 5 presents the corresponding silhouette–Kano visualization. The network included 64 publications and 31 coauthors, with a silhouette width of 0.82 and AAC of 0.62.

**Figure 5.**
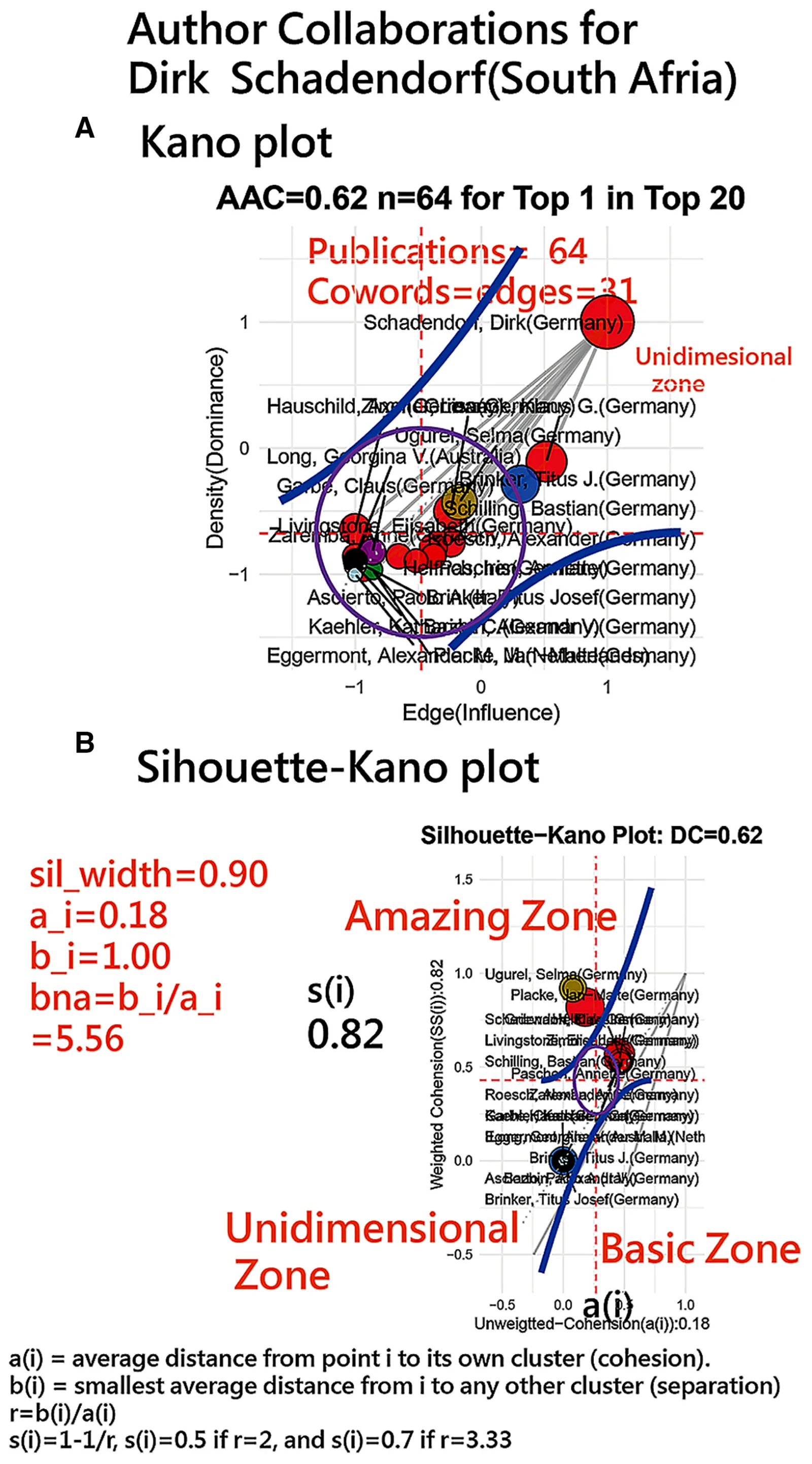
Silhouette–Kano plot for author collaborations of Dr Dirk Schadendorf (rank 6774 among top 2% scientists). This figure integrates silhouette metrics with Kano-style zones to assess collaboration quality. His network (n = 64 publications; 31 coauthors) shows high cohesion (sil_width = 0.82, AAC = 0.62), positioned in the “Amazing Zone,” indicating strong dominance and group synergy. AAC = absolute advantage coefficient.

### 3.4. Profile and collaboration network of Michael R. Hamblin

Figure 6 summarizes the bibliometric profile of Dr Michael R. Hamblin, including 862 publications and an h-index^[6]^ of 126. The US, Massachusetts General Hospital, Photobiomodulation, Photomedicine, and Laser Surgery, and photomedicine-related research terms were prominently represented in the profile. The overall AAC was 0.77.

**Figure 6.**
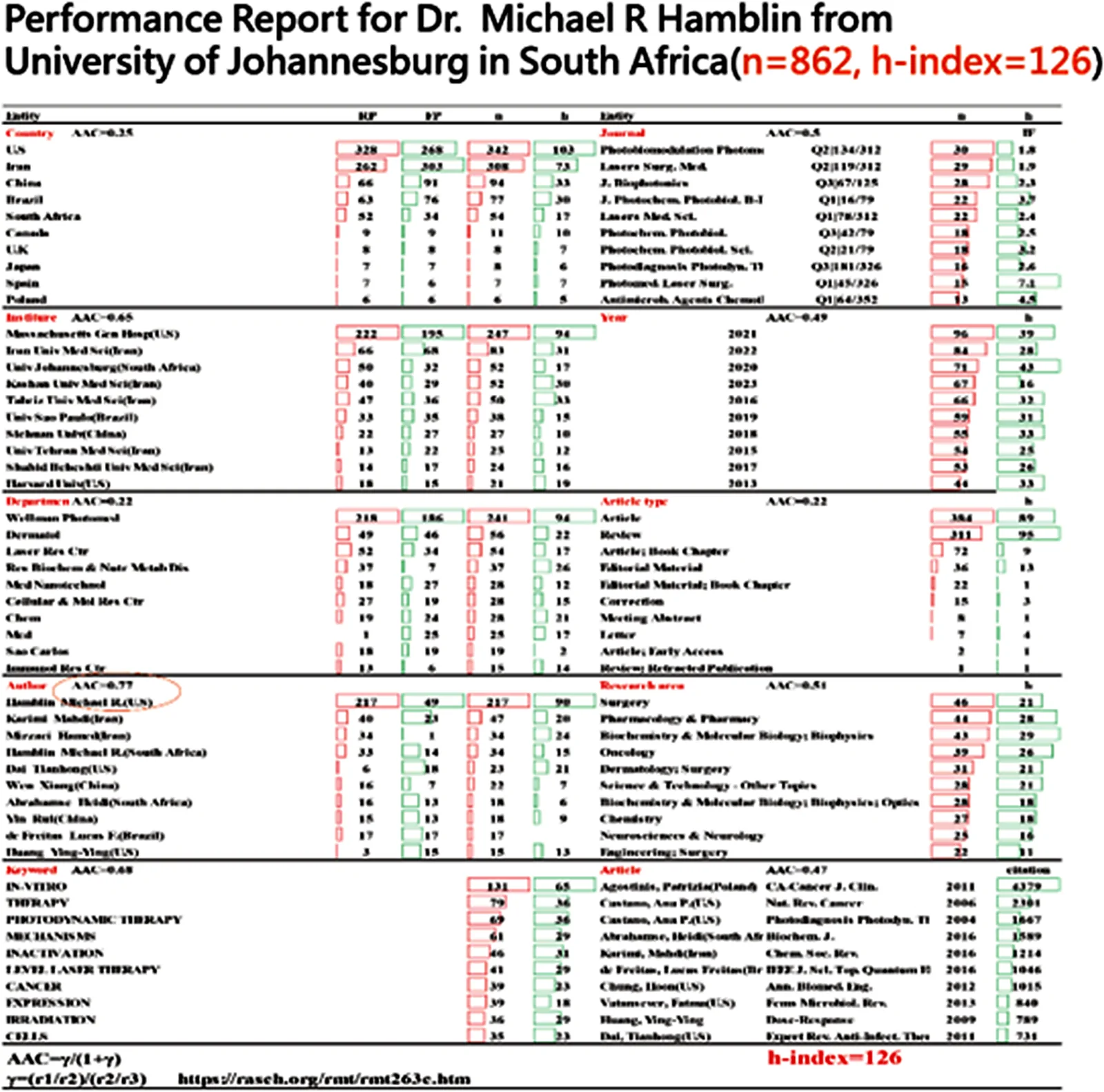
Performance report for Dr Michael R. Hamblin (global rank: 723 among Top 2% scientists). This bibliometric summary (n = 862; h-index = 126) emphasizes contributions from the US and Massachusetts General Hospital in photobiomodulation and photomedicine research. AAC = 0.77 identifies his leadership in phototherapy and laser medicine. AAC = absolute advantage coefficient, US = United States.

Figure 7 presents Dr Hamblin coauthor network, comprising 72 nodes and 13 clusters. The overall SS was 0.71, Q* was 0.74, and AAC was 0.76. Dai Tianhong, Ebrahimi Nasim, and Abrahamsse Heidi were among the identified collaborators.

**Figure 7.**
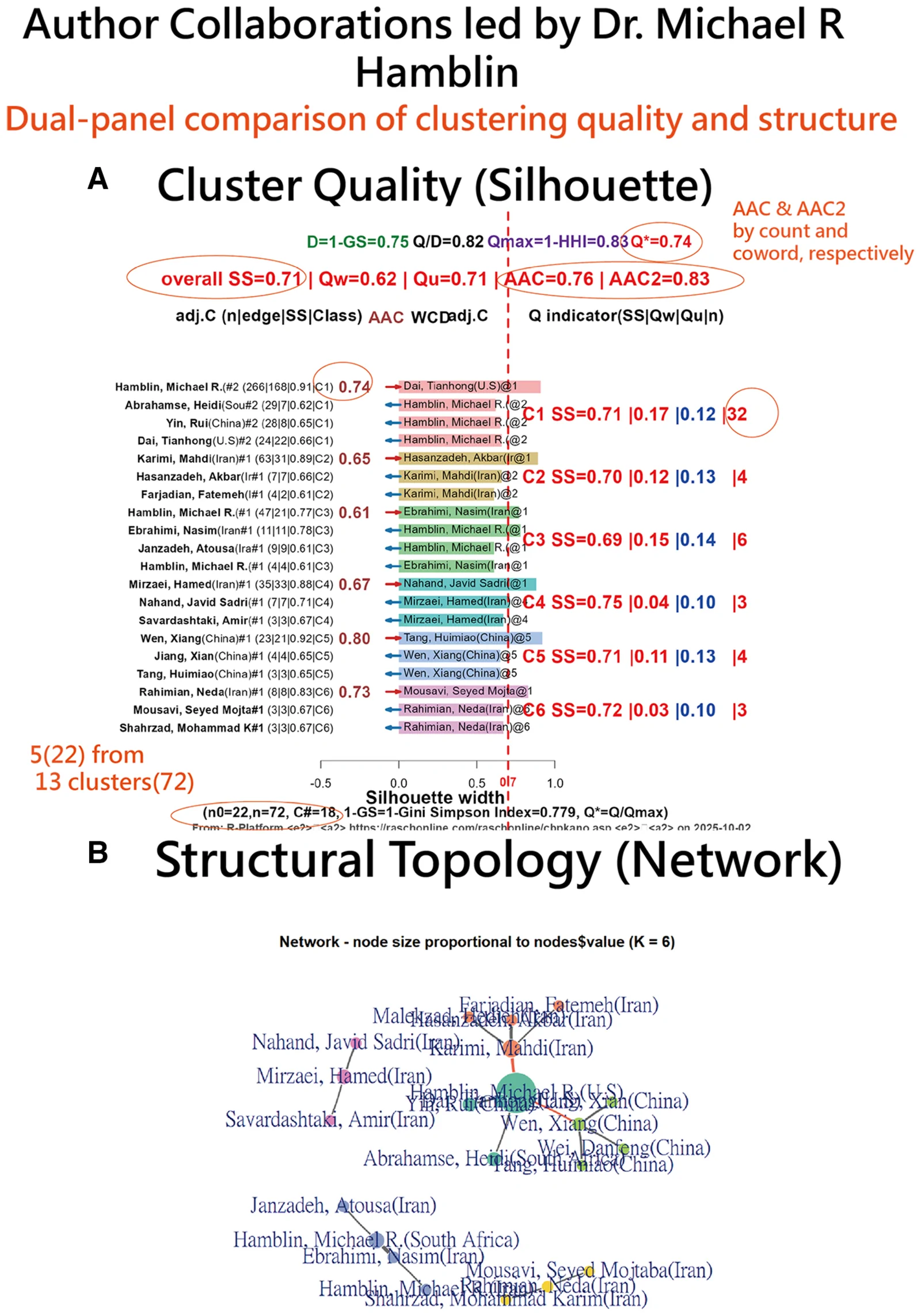
Silhouette plot of author collaborations led by Dr Michael R. Hamblin (rank 723 among top 2% scientists). This dual-panel visualization of collaboration clusters (n = 72; k = 13) shows the silhouette panel (top) indicating strong cluster quality (overall SS = 0.71, Q* = 0.74, AAC = 0.76), while the network panel (bottom) maps global coauthor relationships with collaborators such as Dai Tianhong, Ebrahimi Nasim, and Abrahamsse Heidi. AAC = absolute advantage coefficient, SS = silhouette scores.

Figure 8 presents the corresponding silhouette–Kano visualization. The network included 217 publications and 168 coauthors, with a silhouette width of 0.90 and AAC of 0.77.

**Figure 8.**
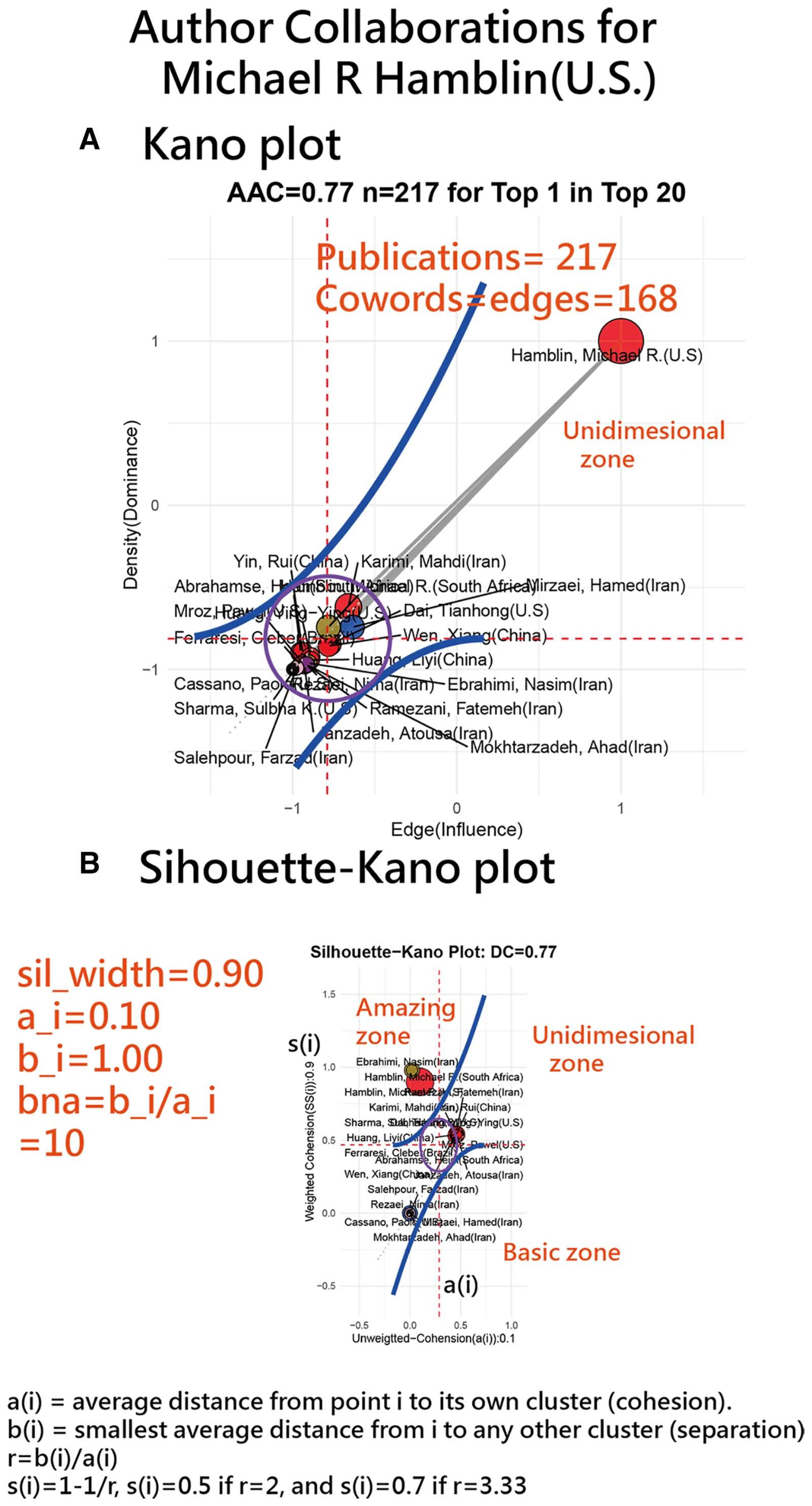
Silhouette–Kano plot for author collaborations of Dr Michael R. Hamblin (rank 723 among top 2% scientists). This figure integrates silhouette metrics with Kano zones to evaluate collaboration strength. The network (n = 217 publications; 168 coauthors) shows high cohesion (sil_width = 0.90; AAC = 0.77), located in the “Amazing Zone,” reflecting exceptional leadership and multidimensional teamwork in photomedicine. AAC = absolute advantage coefficient.

### 3.5. Generation of Figures 1 to 8

The R scripts used to produce Figures 1–8^[43,44,47]^ are summarized in Table 2, along with an accompanying video demonstration.^[48]^ Details are deposited in references^[36,37]^ and GitHub,^[41,42]^ respectively.

**Table 2:** Making Figures 1–8 with R scripts.

Figure	Data type	R-code	Description
3, 6	MetaData	^[19]^	Summary report
1, 4, 7	Co-word set	^[43]^	SS plot & network
2, 5, 8	Two axes table	^[44,47]^	Kano plot

SS = silhouette scores.

## 4. Discussions

### 4.1. Principal findings

This study analyzed 463 top 2% dermatology scientists from 35 countries and 360 institutions, identifying 15 major clusters. The US, Germany, the UK, Australia, and Sweden were the most prominent countries, while Harvard Medical School, Charité–Universitätsmedizin Berlin, and the University of California, San Francisco emerged as major hubs. Dirk Schadendorf was the most cited scholar, whereas Michael R. Hamblin ranked highest in dermatology. Overall, SS, Q*, and AAC provided complementary information on local cluster separation, global community structure, and leadership concentration, respectively (Table 3).

**Table 3:** Summary of study findings across the 3 analytic components and their interpretations.

Research component	Metric	Question answered in discussion	Interpretation
Local cluster separation	SS	Are individual clusters clearly separated?	Higher SS → stronger cohesion/separation
Global community structure	Q*	Is the network as a whole organized into robust communities?	Higher Q* → stronger global community organization
Leadership concentration	AAC	Is scholarly contribution concentrated around 1 leader or distributed among several cores?	Higher AAC → greater leadership concentration
Joint visualization	Amazing zone	Which entities perform strongly on both plotted dimensions?	Descriptive high–high region
Country/institution patterns	Network structure	Where are major hubs and bridges located?	Structural prominence, not necessarily quality
Schadendorf vs Hamblin	AAC + citation/ranking	Do impact, ranking, and leadership concentration tell the same story?	No; they represent distinct dimensions

AAC = absolute advantage coefficient, SS = silhouette scores.

### 4.2. Interpretation of the network patterns

The country- and institution-level findings also provide substantive information about the organization of dermatology research. The prominence of the US, Germany, the UK, Australia, and Sweden suggests that internationally influential dermatology research remains concentrated within a relatively limited number of national research systems. At the institutional level, the emergence of Harvard Medical School, Charité–Universitätsmedizin Berlin, and the University of California, San Francisco as major hubs indicates that a small number of institutions occupy structurally important positions within the collaboration network.

The largest cluster, led by the US, contained the greatest number of observed nodes and showed extensive cross-linkages with other clusters. This pattern suggests that the US functions not only as a large contributor but also as an important connector among institutional and national research communities. Strong weighted connections between clusters may therefore reflect the role of major research systems in linking otherwise more specialized communities. Nevertheless, these network patterns should not automatically be interpreted as evidence of superior research quality or causal influence because network prominence can also be affected by publication volume, database coverage, collaboration practices, and the construction of the network itself.

The joint visualization of the indicators further produced an upper-right “Amazing Zone.” In this study, the Amazing Zone refers to the region in which entities simultaneously show high values on the 2 dimensions displayed in a given scatterplot; for example, high cluster quality together with strong structural prominence or high leadership concentration. Entities located in this region therefore satisfy 2 desirable network characteristics simultaneously rather than excelling on only 1 dimension. The term should consequently be understood as a descriptive visualization aid rather than as a formally validated statistical classification.

Clarivate Analytics annually publishes a list of the most influential scientists of the past decade. However, its classification system is broad (covering only 21 fields and including approximately 6000 scientists^[49]^) and does not provide visual analyses integrating silhouette plots with network graphs.

Traditional author publication lists and bibliometric analyses have several limitations that remain unaddressed. For instance, excessive figures and tables (e.g., 42 in 1 paper^[50]^) often overwhelm readers and fail to concisely convey major contributions or relationships, especially regarding the quantitative quality of clusters.

In contrast, the observed distribution in these plots was characterized by a relatively small number of countries, institutions, or scholars occupying the upper-right region, while many observations were concentrated closer to the origin. Such a distribution is compatible with the well-known Matthew effect,^[51]^ in which visibility, resources, and collaborative opportunities tend to accumulate around already prominent scientific actors. However, the present cross-sectional network analysis cannot establish that cumulative advantage caused the observed distribution.

### 4.3. Illustrative scholar-level findings

Dirk Schadendorf and Michael R. Hamblin were particularly informative illustrative cases because they represented different dimensions of bibliometric prominence. Schadendorf was identified as the most highly cited scholar in the dataset, whereas Hamblin ranked highest within the dermatology field. Their comparison therefore demonstrates that citation impact, field-specific ranking, and collaboration leadership concentration are related but conceptually distinct dimensions of scholarly performance.

Their AAC values further illustrate this distinction. If the values reported in the Results are approximately 0.66 for Schadendorf and 0.76 for Hamblin, they fall into different categories under the Table 1 classification. An AAC of approximately 0.66 is consistent with a more distributed, multicore collaboration structure, whereas an AAC of approximately 0.76 indicates substantially greater concentration around a single leading contributor. Thus, a researcher with the greatest citation impact does not necessarily display the highest degree of leadership concentration.

These examples demonstrate the additional information provided by AAC beyond traditional citation indicators. Rather than simply identifying who has accumulated the largest number of citations, AAC characterizes how strongly scholarly contributions are concentrated around the focal investigator within the observed collaboration network. Such information may be relevant when considering academic leadership and the structural conditions under which junior researchers participate in research teams.

Importantly, however, leadership concentration should not be equated with mentorship. A high-AAC value indicates concentration of authorship or collaborative contribution around a focal scholar, but it does not establish that the scholar actively trained, supervised, or mentored junior researchers. Conversely, a lower AAC value may represent distributed or team-based leadership rather than weak mentorship. Direct assessment of mentorship would require additional information such as advisor–trainee relationships, career stage, supervision records, longitudinal coauthorship trajectories, or documented training activities.

### 4.4. Methodological contribution and implications

Previous analyses of the world’s top 2% scientists^[10]^ have primarily emphasized citation-based rankings, including the *h*-index^[6]^ and composite *c*-score. Such indicators are useful for assessing cumulative scholarly impact but provide limited information about the structural organization of research communities or the distribution of leadership within collaboration networks.

The present framework addresses this limitation by integrating 3 complementary indicators into a common visualization workflow. SS provides a quantitative assessment of local cluster separation, (Q*) evaluates global community structure, and AAC characterizes leadership concentration. The contribution of the study therefore lies not in proposing a new clustering algorithm^[16,30,39,40]^ or a new bibliometric indicator, but in combining established quantitative measures that are usually reported separately and linking them to interpretable network and profile visualizations.

The normalized modularity measure (Q*=Q/Q_max_) is particularly useful because it expresses modularity relative to its attainable maximum under the specified network setting, facilitating interpretation of global community structure. In parallel, silhouette plots^[36]^ allow readers to visually associate individual nodes and clusters with quantitative measures of cluster separation. AAC adds a further layer by showing whether scholarly contributions are distributed among several major contributors or concentrated around a dominant investigator.

From a research-management perspective, these complementary dimensions may provide more informative profiles than citation counts alone. Institutions could identify well-separated research specialties, examine how strongly research communities are interconnected, and characterize whether collaboration structures depend heavily on a few leading scholars or are distributed across multiple cores. High-AAC groups may warrant further investigation of succession planning, team development, or opportunities for junior investigators, whereas multicore groups may reflect more distributed leadership structures. Such interpretations, however, should be treated as hypotheses for management assessment rather than conclusions about actual mentorship performance.

Because the workflow uses routinely available top 2% scientists data^[10]^ and documented analytic procedures, it can be repeated across disciplines and future annual releases. Longitudinal applications could determine whether community structures become more or less differentiated, whether institutional hubs change over time, and whether leadership concentration increases or decreases within research teams.

### 4.5. Limitations and future research

Several limitations should be considered when interpreting these findings.

First, citation-based indicators such as the *h*-index^[6]^ and composite *c*-score inherently favor researchers with longer publication histories and may therefore underrepresent rapidly emerging early-career scientists.

Second, citation counts do not necessarily reflect research quality because they are affected by field size, topic popularity, publication age, self-citation, and collaboration patterns.

Third, the principal sample was derived from a single source, the Elsevier/Stanford top 2% scientists database.^[10]^ Although this database provides standardized cross-field indicators, alternative databases such as Web of Science (WoS), Google Scholar, and PubMed may yield different coverage and network structures.

Fourth, author-level publication information was obtained from WoS to identify authorship positions and corresponding authors. Differences in author identification, publication coverage, and metadata across WoS and Scopus may introduce inconsistencies.

Fifth, although SS, (Q*), and AAC quantify complementary aspects of network structure, their interpretation depends on network-construction rules, similarity measures, clustering algorithms,^[16,30,39,40]^ threshold settings, and data-cleaning procedures. Comparisons across disciplines or years should therefore employ consistent analytic protocols.

Sixth, country and institutional networks were constructed using co-word or relational similarity and should be interpreted as approximations of research associations rather than direct measures of organizational collaboration. In addition, only the most prominent nodes and clusters were displayed because of visualization constraints.

Most importantly, AAC measures concentration within the observed collaboration structure and is not direct evidence of mentor–mentee relationships. High leadership concentration may be compatible with strong supervision of junior researchers, but it may also arise from authorship conventions, laboratory organization, research specialization, or repeated collaboration among established investigators. Likewise, distributed leadership does not imply weak mentorship. Future studies should combine AAC with researcher career stage, first and corresponding-author transitions, advisor–trainee information, longitudinal coauthorship data, and subsequent independent publication records to determine whether leadership concentration is genuinely associated with mentoring outcomes.

Future research may also examine whether scholars or institutions that remain within the “Amazing Zone” over multiple years subsequently demonstrate stronger research continuity, broader international collaboration, or greater development of independent junior investigators. Such longitudinal validation would transform the current descriptive visualization framework into a stronger tool for evaluating changes in research-community structure and academic leadership.

## 5. Conclusion

This study demonstrates an integrated framework combining SS, normalized modularity (Q*), and AAC to evaluate local cluster separation, global community structure, and leadership concentration among 2025 top 2% dermatology scientists. The analyses identified dominant countries, hub institutions, and representative scholars, while distinguishing citation impact from collaboration leadership patterns. The framework is reproducible and interpretable and may support comparative assessment, research mapping, and performance monitoring across disciplines, although leadership concentration should not be interpreted as direct evidence of mentorship.

## Acknowledgments

We thank Enago (www.enago.tw) for the English language review of this manuscript.

## Author contributions

**Conceptualization:** Yu-An Wang.

**Data curation:** Yu-An Wang, Po-Chih Lai.

**Formal analysis:** Po-Chih Lai.

**Investigation:** Feng-Jie Lai.









## References

[1] Ioannidis JPA Baas J Klavans R Boyack KW. A standardized citation metrics author database annotated for scientific field. PLoS Biol. 2019;17:e3000384. 10.1371/journal.pbio.3000384 PMC6699798 31404057

[2] Yeung AWK Tsoi JKH. A critical correlate of QS subject ranking and stanford’s top 2% scientists in top dental schools. Int Dent J. 2025;75:100954. 10.1016/j.identj.2025.100954 PMC12361774 40811959

[3] Abdalla BA Mustafa AM Fattah FH. Self-citation pattern among world’s top 2 % of the scientists. Heliyon. 2025;11:e42471. 10.1016/j.heliyon.2025.e42471 PMC11849628 39995918

[4] Ioannidis JPA Boyack KW Baas J. Updated science-wide author databases of standardized citation indicators. PLoS Biol. 2020;18:e3000918. 10.1371/journal.pbio.3000918 PMC7567353 33064726

[5] Wu AL Chou W. Identifying China’s distinctive academic fields among the top 2% of influential scientists: a bibliometric analysis using Rasch KIDMAP. Medicine (Baltimore). 2024;103:e36706. 10.1097/MD.0000000000036706 PMC10766269 38181244

[6] Hirsch JE. An index to quantify an individual’s scientific research output. Proc Natl Acad Sci U S A. 2005;102:16569–72. 10.1073/pnas.0507655102 PMC1283832 16275915

[7] Walach H. Complementary medicine has reached the centre of scientific interest: some thoughts on ioannidis’ citation database. Complement Med Res 2019;26:373–5. 10.1159/000504467 31739304

[8] Van Noorden R Singh Chawla D. Hundreds of extreme self-citing scientists revealed in new database. Nature. 2019;572:578–9. 10.1038/d41586-019-02479-7 31455906

[9] Ioannidis JP Klavans R Boyack KW. Multiple citation indicators and their composite across scientific disciplines. PLoS Biol. 2016;14:e1002501. 10.1371/journal.pbio.1002501 PMC4930269 27367269

[10] Elsevier Data Repository. August 2024 data-update for “Updated science-wide author databases of standardized citation indicators.” 2025. Available at https://elsevier.digitalcommonsdata.com/datasets/btchxktzyw/7. Accessed September 28, 2026. 10.1371/journal.pbio.3000918 PMC7567353 33064726

[11] Senior M Fazel S. Measuring an individual researcher’s impact: new directions and challenges. Evid Based Ment Health. 2020;23:47–51. 10.1136/ebmental-2019-300122 PMC10231616 31801786

[12] Slone RM. Coauthors’ contributions to major papers published in the AJR: frequency of undeserved coauthorship. AJR Am J Roentgenol. 1996;167:571–9. 10.2214/ajr.167.3.8751654 8751654

[13] Zbar A Frank E. Significance of authorship position: an open-ended international assessment. Am J Med Sci. 2011;341:106–9. 10.1097/MAJ.0b013e3181f683a1 20924283

[14] Jian D Xiaoli T. Perceptions of author order versus contribution among researchers with different professional ranks and the potential of harmonic counts for encouraging ethical co-authorship practices. Scientometrics. 2013;96:277–95.

[15] Costas R Bordons M. Do age and professional rank influence the order of authorship in scientific publications? Some evidence from a micro-level perspective. Scientometrics. 2011;88:145–61. 10.1007/s11192-011-0368-z PMC3111668 21765565

[16] Chien TW Chou W. Visualizing leadership classifications in rectangular data using a basket model and co-word network analysis: a case study of U.S. HCAHPS survey results. BMC Med Res Methodol. 2025;25:195. 10.1186/s12874-025-02643-w PMC12366396 40836275

[17] Kao WC Chow JC Chou W Chen L. Discrepancy between countries most mentioned in earthquake-related articles and those most affected by major earthquakes since 1965: a bibliometric analysis. Medicine (Baltimore). 2025;104:e43953. 10.1097/MD.0000000000043953 PMC12459510 40988235

[18] Lim SW Chou W Chen L. SankeyNetwork: a clear and concise visualization tool for bibliometric data. MethodsX. 2025;14:103379. 10.1016/j.mex.2025.103379 PMC12179738 40547885

[19] Chien TW Chou W. 10StepsinExcelforWoS: streamlining bibliometric analysis in web of science using Microsoft excel. SoftwareX. 2025;32:102403.

[20] Chuang HY Chou W. SilhouetteScoreinR: beyond traditional network layouts by leveraging local cohesion and nearest neighbor separation. MethodsX. 2025;15:103622. 10.1016/j.mex.2025.103622 PMC12466245 41018252

[21] Blondel VD Guillaume JL Lambiotte R Lefebvre E. Fast unfolding of communities in large networks. J Stat Mech. 2008;2008:P10008.

[22] Callon M Courtial JP Laville F. Co-word analysis as a tool for describing the network of interactions between basic and technological research: the case of polymer chemsitry. Scientometrics. 1991;22:155–205.

[23] van Eck NJ Waltman L. Software survey: VOSviewer, a computer program for bibliometric mapping. Scientometrics. 2010;84:523–38. 10.1007/s11192-009-0146-3 PMC2883932 20585380

[24] Newman MEJ. The structure of scientific collaboration networks. Proc Natl Acad Sci U S A. 2001;98:404–9. 10.1073/pnas.021544898 PMC14598 11149952

[25] Perozzi B Al-Rfou R Skiena S. DeepWalk: Online learning of social representations. In: Proceedings of the 20th ACM SIGKDD International Conference on Knowledge Discovery and Data Mining. New York, NY: Association for Computing Machinery (ACM); 2014:701–10.

[26] Grover A Leskovec J. node2vec: scalable feature learning for networks. In: Proceedings of the 22nd ACM SIGKDD International Conference on Knowledge Discovery and Data Mining. San Francisco, CA: Association for Computing Machinery (ACM); 2016:855–64. 10.1145/2939672.2939754 PMC5108654 27853626

[27] Kipf TN Welling M. Semi-supervised classification with graph convolutional networks. In: 5th International Conference on Learning Representations (ICLR 2017). Toulon, France: OpenReview.net; 2017.

[28] Mikolov T Chen K Corrado G Dean J. Efficient estimation of word representations in vector space. arXiv. 2013;1301.3781.

[29] Rousseeuw PJ. Silhouettes: A graphical aid to the interpretation and validation of cluster analysis. J Comput Appl Math. 1987;20:53–65.

[30] Li MJ Lai FJ. Classifying research leadership in infantile hemangioma studies: a bibliometric analysis using Kano diagrams and absolute advantage coefficients. Medicine (Baltimore). 2025;104:e44905. 10.1097/MD.0000000000044905 PMC12499838 41054051

[31] Yang DH Chien TW Yeh YT Yang T-Y Chou W Lin J-K. Using the absolute advantage coefficient (AAC) to measure the strength of damage hit by COVID-19 in India on a growth-share matrix. Eur J Med Res. 2021;26:61. 10.1186/s40001-021-00528-4 PMC8223180 34167582

[32] Yang TY Chien TW Lai FJ. Citation analysis of the 100 top-cited articles on the topic of hidradenitis suppurativa since 2013 using Sankey diagrams: Bibliometric analysis. Medicine (Baltimore). 2022;101:e31144. 10.1097/MD.0000000000031144 PMC9646634 36343026

[33] Newman ME. Modularity and community structure in networks. Proc Natl Acad Sci U S A. 2006;103:8577–82. 10.1073/pnas.0601602103 PMC1482622 16723398

[34] Chen C. CiteSpace II: detecting and visualizing emerging trends and transient patterns in scientific literature. J Am Soc Inf Sci. 2005;57:359–77.

[35] Newman MEJ Girvan M. Finding and evaluating community structure in networks. Phys Rev E Stat Nonlin Soft Matter Phys. 2004;69:026113. 10.1103/PhysRevE.69.026113 14995526

[36] Chien TW Chen L Chou W. SilhouettePlot: an R-based app integrating silhouette scores into network plots for enhanced clarity and meaning. SoftwareX. 2026;34:102652.

[37] Chien TW Chen L Chou W. ClusterChoice: a web-based tool for interpretable clustering and algorithm comparison in bibliometric networks. SoftwareX. 2026;35:102794.

[38] Chien TW. Dimension coefficient for evaluating unidimensionality: interpreting PCA of standardized residuals in RaschOnline. Rasch Meas Trans. 2026;38:2026–32.

[39] Cheng YZ Chien TW Ho SY Chou W. Visual impact beam plots: analyzing research profiles and bibliometric metrics using the following-leading clustering algorithm (FLCA). Medicine (Baltimore). 2023;102:e34301. 10.1097/MD.0000000000034301 PMC10344504 37443470

[40] Yen PC Chou W Chien TW Jen TH. Analyzing fulminant myocarditis research trends and characteristics using the follower-leading clustering algorithm (FLCA): a bibliometric study. Medicine (Baltimore). 2023;102:e34169. 10.1097/MD.0000000000034169 PMC10313307 37390236

[41] Github. SilhouettePlot with R scripts. 2026. Available at :https://github.com/smilechien/clusterchoice/. Accessed September 28, 2026.

[42] Github. FLCA clustering algorithm with R scripts. 2026. Available at: https://github.com/smilechien/clusterchoice/. Accessed September 28, 2026.

[43] R-platform. Silhouette pot. 2026. Available at: https://raschonline.com/raschonline/cbpkano.asp?cbp=FLCA2allfiles. Accessed September 28, 2026.

[44] R-platform. Simple Kano plot. 2026. Available at: https://raschonline.com/raschonline/cbp.asp?cbp=SimpleIPA-KanoDiagarm. Accessed September 28, 2026.

[45] R Core Team. R: A Language and Environment for Statistical Computing. 2026. Available at: https://www.R-project.org/

[46] Posit, PBC. RStudio: Integrated Development Environment for R. Posit Software, PBC; 2022.

[47] R-platform. Coword Kano plot. 2026. Available at: https://raschonline.com/raschonline/cbpgeo.asp?cbp=FLCAalgorithmexample53. Accessed September 28, 2026.

[48] R-platform. Making silhouette plot. 2026. Available at: https://youtu.be/ky362iLoVS0. Accessed September 28, 2026.

[49] WoS. Highly cited researchers 2023. 2026. Available at: https://clarivate.com/academia-government/blog/highly-cited-researchers-2023-the-evolution-of-our-evaluation-and-selection-policy-to-support-a-robust-scholarly-landscape/. Accessed September 21, 2025.

[50] Radu AF Bungau SG Negru PA Marcu MF Andronie-Cioara FL. In-depth bibliometric analysis and current scientific mapping research in the context of rheumatoid arthritis pharmacotherapy. Biomed Pharmacother. 2022;154:113614. 10.1016/j.biopha.2022.113614 36058148

[51] Merton RK. The Matthew effect in science. The reward and communication systems of science are considered. Science. 1968;159:56–63. 5634379

