# Supplementary material for: Mapping local cluster separation, global community structure, and leadership concentration among the 2025 top 2% dermatology scientists: Beyond rankings
Source: Medicine (Baltimore). 2026 Oct 9;105(41):e50851. doi: 10.1097/MD.0000000000050851 (PMC13657522; doi:10.1097/MD.0000000000050851)
Supplement: Supplementary file 1 [file medi-105-e50851-s003.pdf]

Elsevier Data Repository. August 2024 data-update for "Updated science-wide author databases of standardized citation. 2025/9/20 at https://elsevier.digitalcommonsdata.com/datasets/btchxktzyw/7

	A	B	C	D	E	F	G	H	I	J	
1	authfull	inst_name	cntry	np6023	firstyr	lastyr	rank (ns)	nc9623 (ns	h23 (ns)	hm23 (ns)	np
2	Hamblin, Michael	University of Johan	zaf	1002	1975	2024	723	59955	118	57.97492	
3	Watt, Fiona M.	European Molecula	deu	433	1978	2024	1569	37490	106	52.71598	
4	Robert, Caroline	Institut de Cancerol	fra	563	1990	2024	1743	100216	124	26.45185	
5	Sen, Chandan K.	Indiana University S	usa	412	1990	2021	2281	26631	79	41.01523	
6	Potten, Christophe	Epistem Limited	gbr	367	1968	2019	3327	21052	79	41.14491	
7	Rohrich, Rod J.	Dallas Plastic Surge	usa	1042	1983	2024	3927	24827	79	46.62168	
8	Ghannoum, Mahn	Case Western Reser	usa	446	1981	2024	5642	25919	81	34.54932	
9	Schadendorf, Dirk	Universitätsklinikur	deu	1130	1989	2024	6774	123547	142	33.94214	
10	Garbe, Claus	Universitätsklinikur	deu	813	1985	2025	7233	52957	104	34.0682	
11	Wick, Mark R.	University of Virgir	usa	645	1980	2024	7340	18982	75	39.98961	
12	Dummer, Reinhar	UniversitatsSpital Z	che	1024	1988	2024	7475	76056	120	36.068	
13	Modlin, Robert L.	University of Califo	usa	384	1975	2024	7562	38348	106	34.54322	
14	Green, A. C.	The University of M	gbr	598	1976	2021	8515	28509	87	39.42736	
15	Suster, Saul	Medical College of	usa	431	1984	2024	9525	16482	65	39.7334	
16	Lussi, Adrian	University of Bern	che	528	1986	2024	9860	17660	66	37.42183	

	A	B	C
1	inst_name	cntry	
2	University of Joha	zaf	
3	European Molecul	deu	
4	Institut de Cancer	fra	
5	Indiana University	usa	
6	Epistem Limited	gbr	
7	Dallas Plastic Surg	usa	
8	Case Western Res	usa	
9	Universitätsklinik	deu	
10	Universitätsklinik	deu	
11	University of Virg	usa	
12	UniversitatsSpital	che	
13	University of Cali	usa	
14	The University of	gbr	

Country.csv (n=463)

https://raschonline.com/raschonline/cbpkano.asp?cbp=FLCA2allfiles

Modularity Qc eqal when not 2

@@@FLCA SS plots to nodes & data all file

Example Qw uqualto Qu

Submit

Tips for R

Copy & Paste the Code in R to Rstudio

Select text

```
#
########################################
 ########################################


# Load the datasets
library(readr)
library(tidyr)
```

```
4      0.7150           0 0.110422 0.16
5      0.6800           0 0.064606 0.16
6      0.7180          NA 0.751418 0.80
>
>               ### past R script to draw SS plot: https://raschonline.com/raschonline/cbpkano.asp?cbp=ModularityQrelationsetmaxTRUE2
>               cat("save png in F:/RR/ and C:\Users\user\Documents\t")
Error: '\U' used without hex digits in character string starting ""save png in F:/RR/ and C:\U"
>               write.csv(nodes,"F:/RR/nodes.csv")
>
>
> nodes
         name value carac value2
1         usa   185     1    185
2  Harvard Me     8     1      8
3  University     5     1      5
4  University     5     1      5
```

Top 2% Scientists(Stanford/Elsevier) in Dermatology & Venereal Diseases 2025

Dual-panel comparison of clustering quality and structure

(A) Cluster Quality (Silhouette)

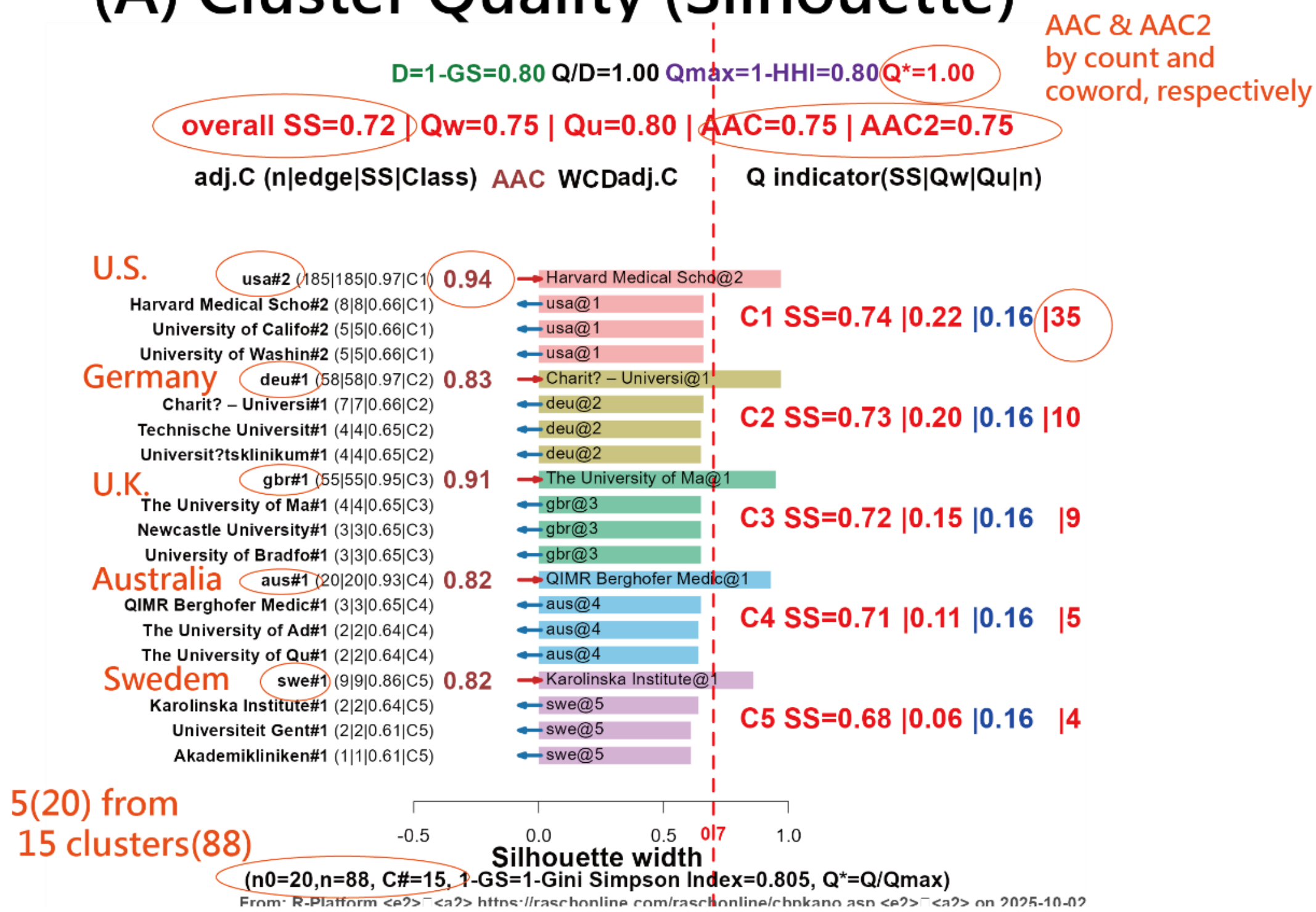

(B) Structural Topology (Network)

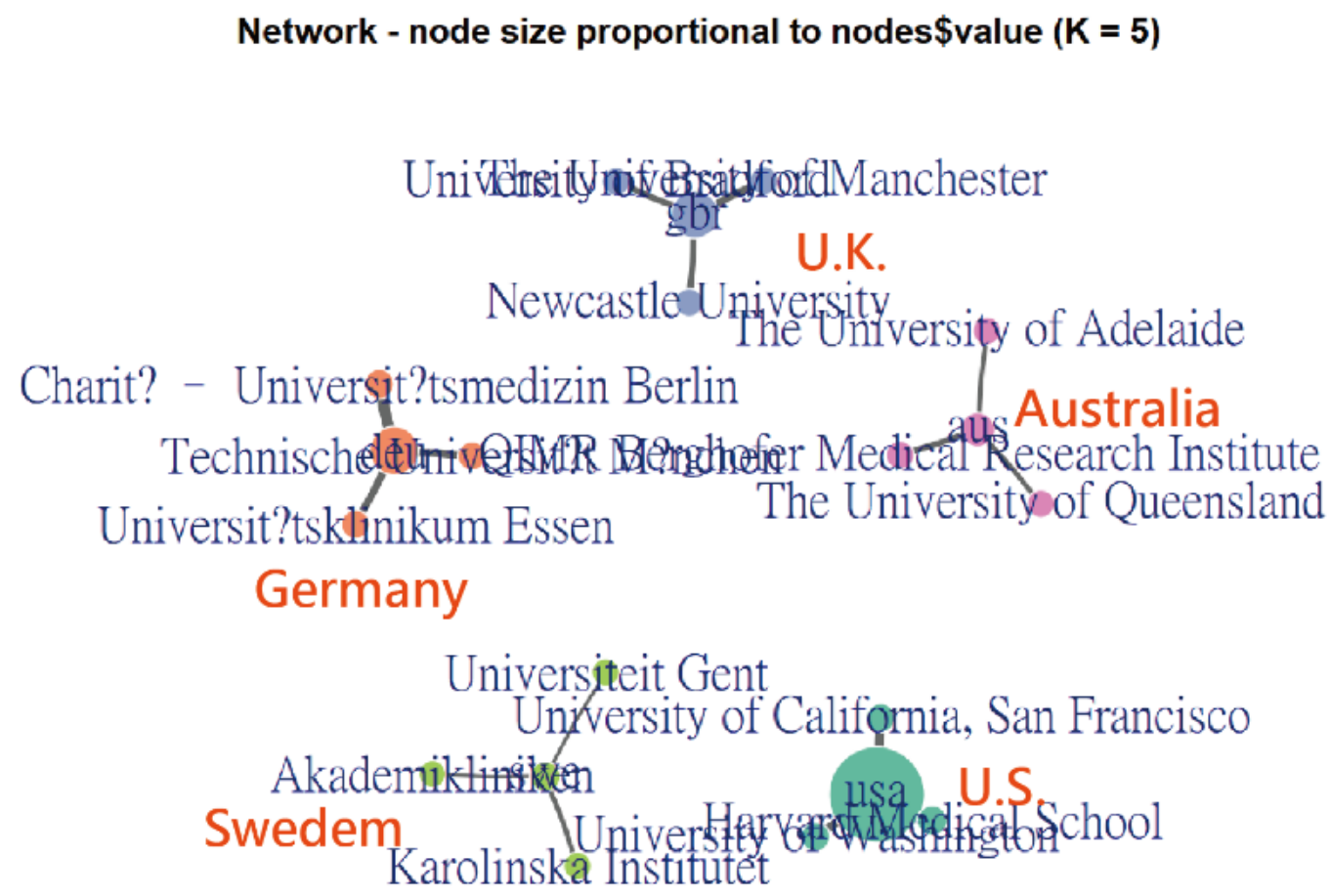

R plate form for simplified Kano plot on 2025/9/25at

https://raschonline.com/raschonline/cbp.asp?cbp=SimpleIPA-KanoDiagarm

	A	B	C	D
1	authfull	np6023	nc9623 (ns	cara
2	Hamblin, Michae	1002	59955	1
3	Watt, Fiona M.	433	37490	1
4	Robert, Caroline	563	100216	1
5	Sen, Chandan K	412	26631	1
6	Potten, Christoph	367	21052	1
7	Rohrich, Rod J.	1042	24827	2
8	Ghannoum, Mah	446	25919	2
9	Schadendorf, Di	1130	123547	2
10	Garbe, Claus	813	52957	2
11	Wick, Mark R.	645	18982	2
12	Dummer, Reinha	1024	76056	2
13	Modlin, Robert I	384	38348	2
14	Green, A. C.	598	28509	2
15	Suster, Saul	431	16482	2
16	Lussi, Adrian	528	17660	2
17	Asero, Riccardo	404	13635	3

```
# Load libraries
library(ggplot2)
library(dplyr)
library(ggrepel)

# Step 0: Create the dataframe
nodes <- read.table(textConnection('
name    value   value2  cluster
Hamblin, Michael R. 1002    59955   1
Watt, Fiona M.  433 37490   1
Robert, Caroline    563 100216  1
Sen, Chandan K. 412 26631   1
Potten, Christopher S.  367 21052   1
Rohrich, Rod J. 1042    24827   2
Ghannoum, Mahmoud   446 25919   2
Schadendorf, Dirk   1130    123547  2
Garbe, Claus    813 52957   2
Wick, Mark R.   645 18982   2
Dummer, Reinhard    1024    76056   2
Modlin, Robert L.   384 38348   2
Green, A. C.    598 28509   2
```

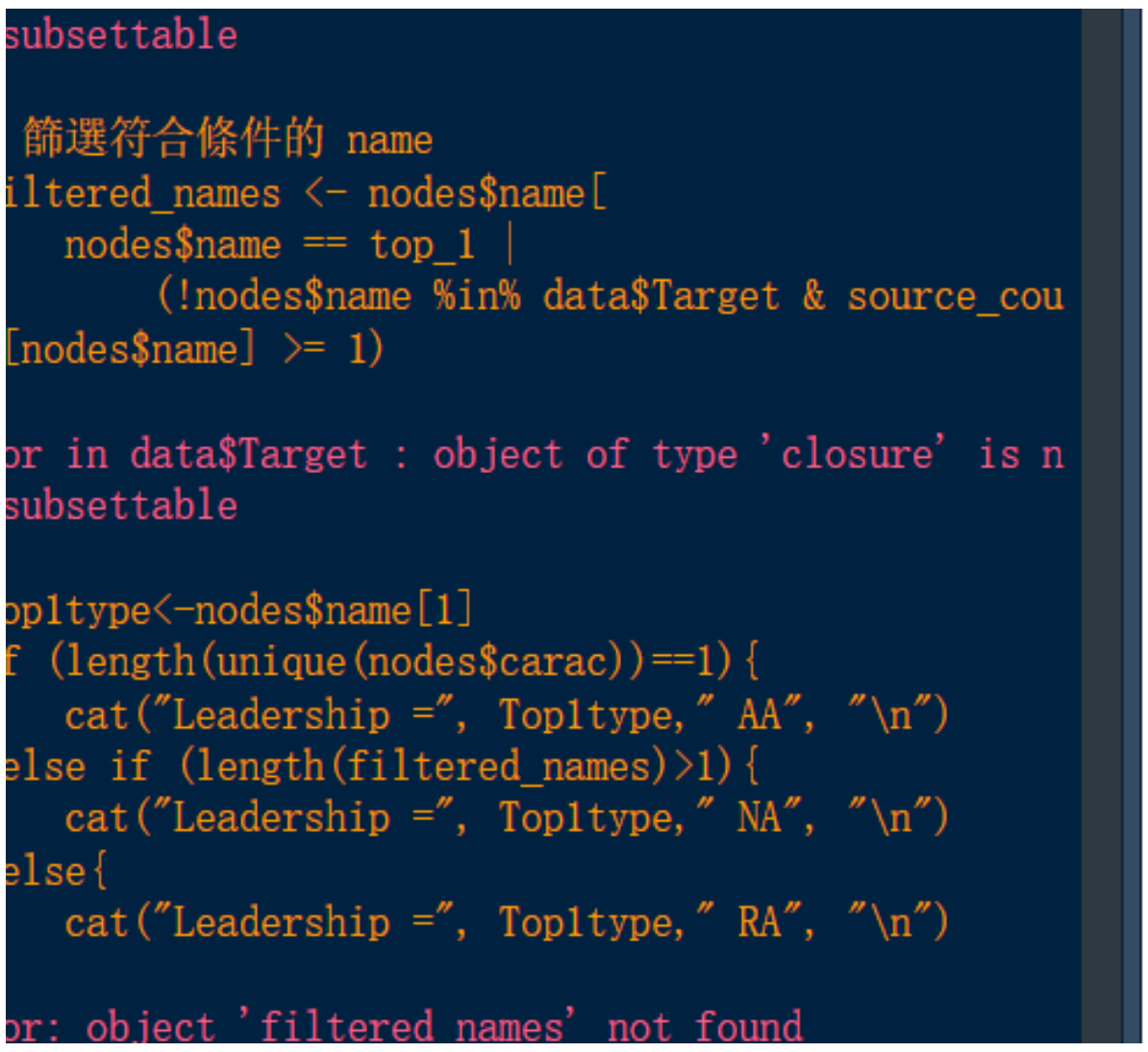
```
subsettable

 篩選符合條件的 name
iltered_names <- nodes$name[
   nodes$name == top_1 |
      (!nodes$name %in% data$Target & source_cou
[nodes$name] >= 1)

or in data$Target : object of type 'closure' is n
subsettable

op1type<-nodes$name[1]
f (length(unique(nodes$carac))==1){
   cat("Leadership =", Top1type," AA", "\n")
else if (length(filtered_names)>1){
   cat("Leadership =", Top1type," NA", "\n")
else{
   cat("Leadership =", Top1type," RA", "\n")

or: object 'filtered_names' not found
```

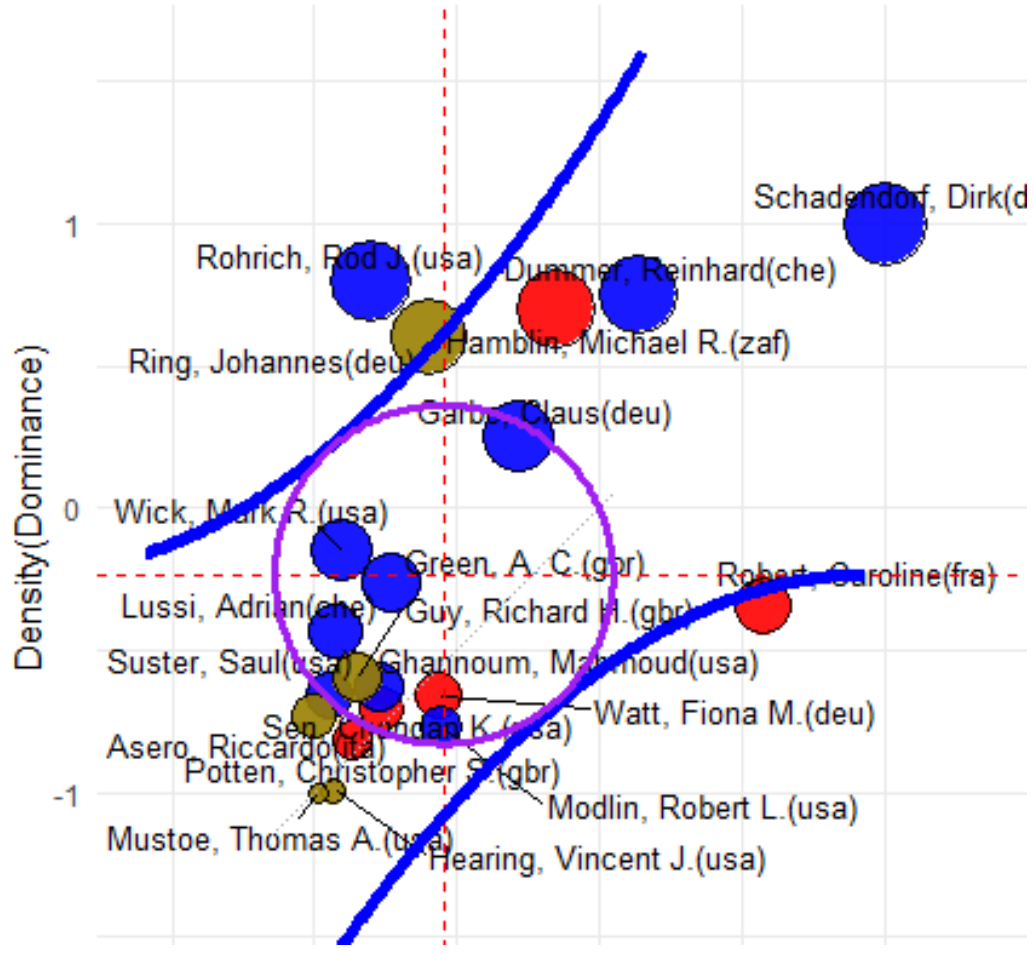

Top 20 Scientists in

Dermatology & Venereal Diseases 2025

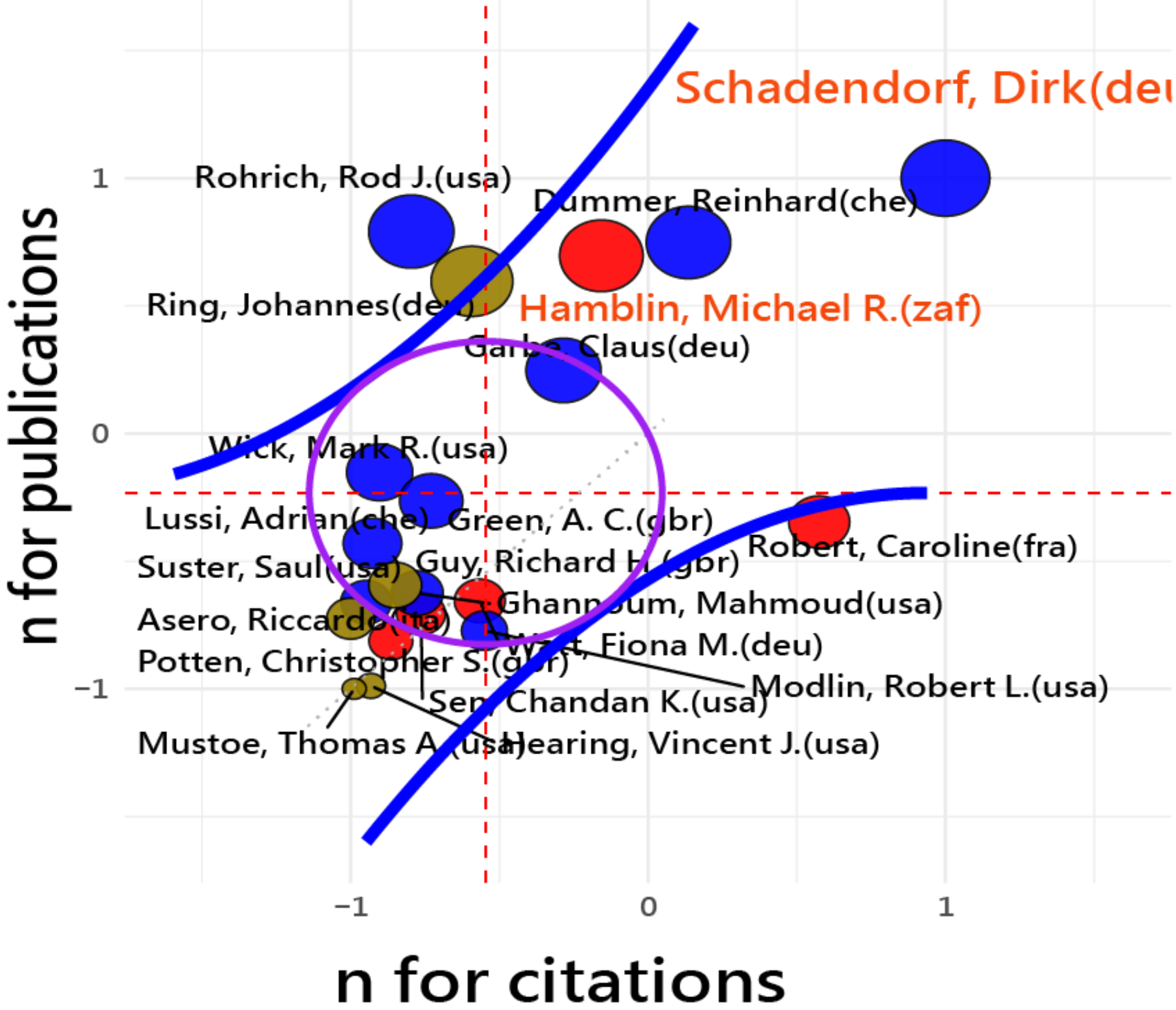

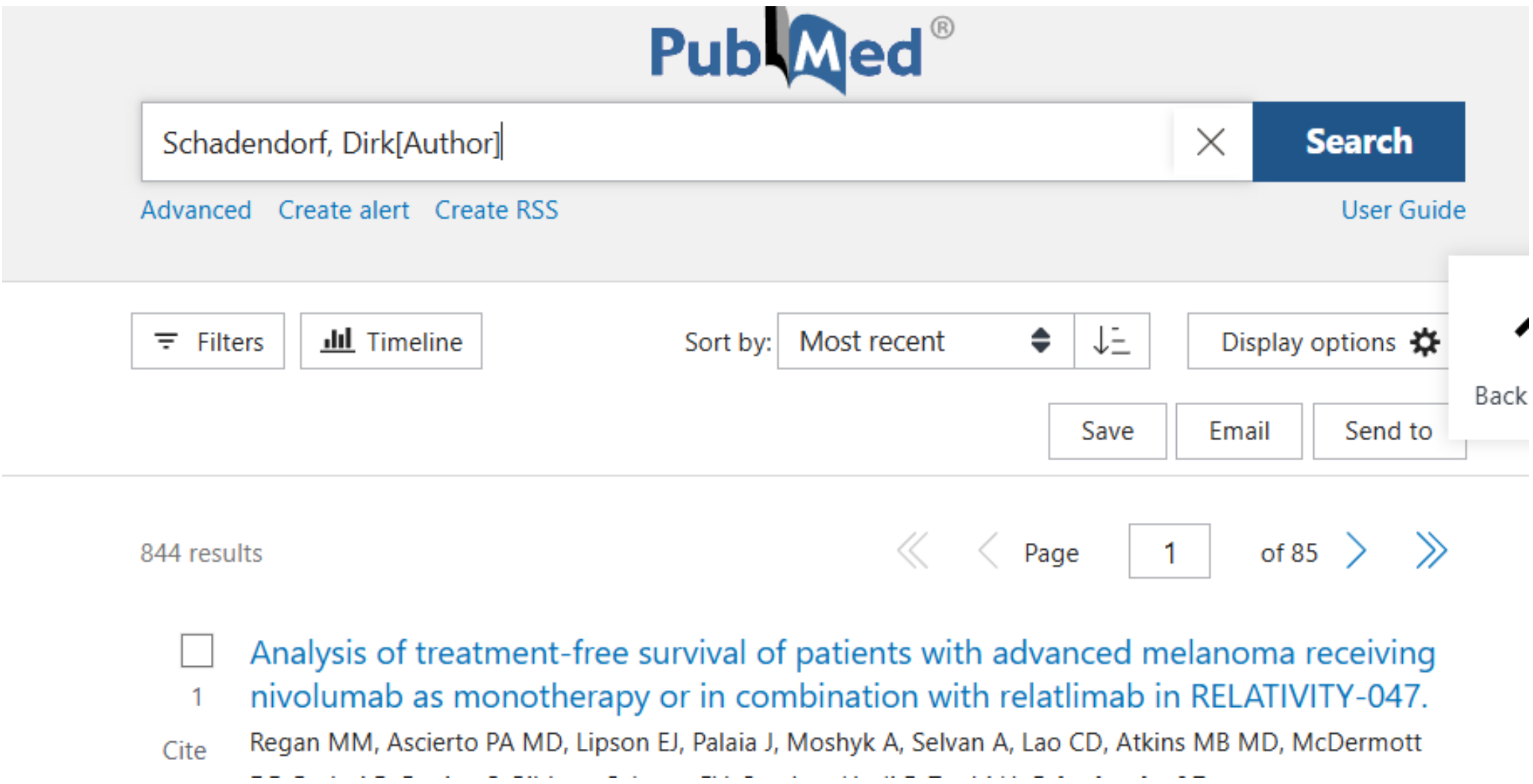

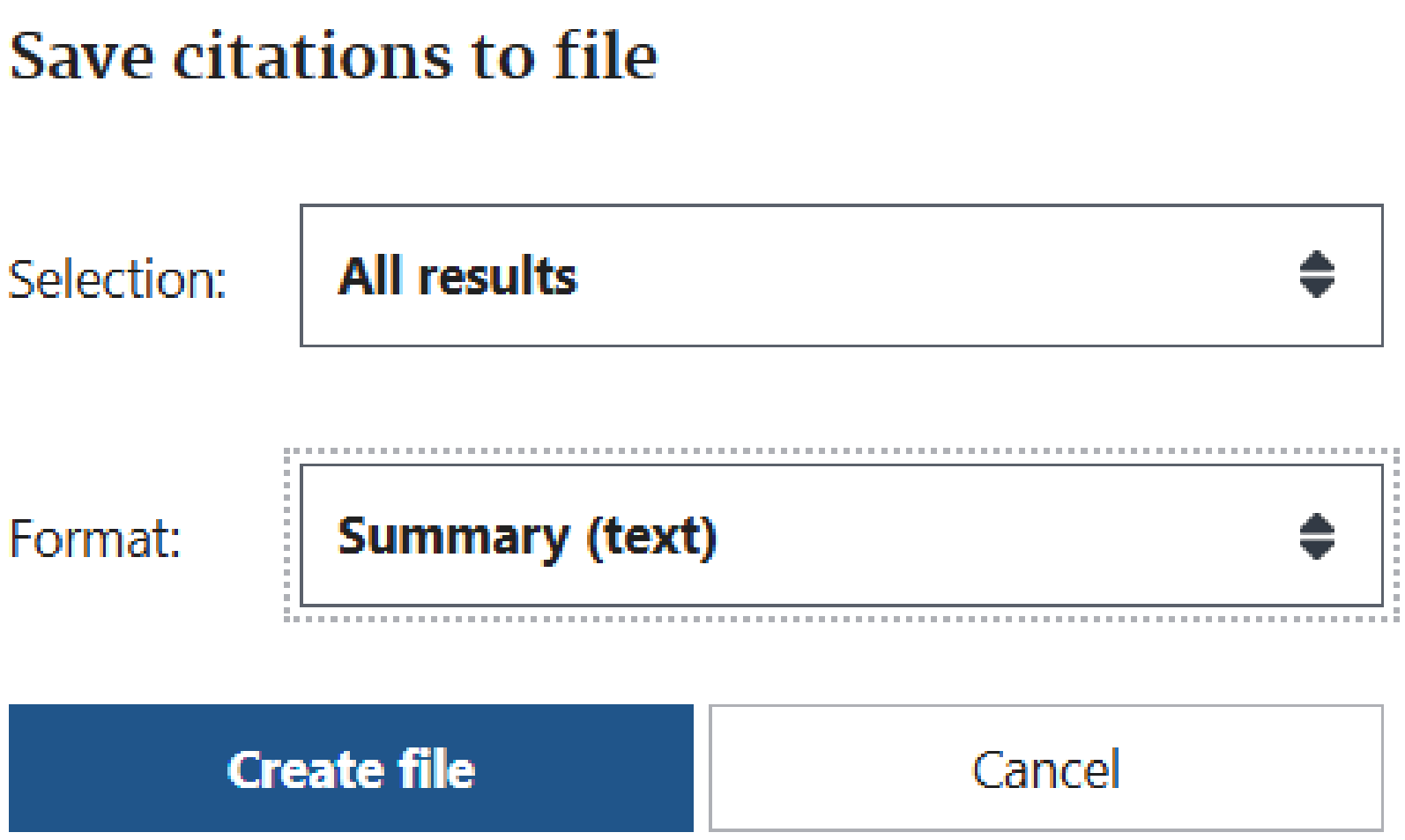

https://raschonline.com/raschonline/cbpkano.asp?cbp=PubMedJouranlNamefromSummaryreference

Modulaity Qc eqal when not 2
@@@FLCA SS plots to nodes & data all file
Example Qw uqualto Qu

Submit

Tips for R

Copy & Paste the Code in R to Rstudio

Select text

```
# for cited reference from https://icite.od.nih.gov/
## ==Sys.setenv(NCBI_API_KEY = "4795cdb815101de551825aa1017a90a0f108")
## Sys.getenv("NCBI_API_KEY")

 ## ===== Config =====
RUN <- FALSE  # TRUE: process file; FALSE: run demo
ref_path <- "C:/Users/user/Downloads/summary-Tsair-WeiC-set.txt"
out_csv  <- paste0(sub("\\.txt$","", ref_path, ignore.case = TRUE), "_journal_authors.cs
v")

## ===== Utils: robust read as UTF-8 =====
read_text_utf8 <- function(path) {
    if (!file.exists(path)) stop("File not found: ", path)
    ln <- readLines(path, encoding = "UTF-8", warn = FALSE)
```

```
"4795cdb815101de551825aa1017a90a0f108")
## Sys.getenv("NCBI_API_KEY")

## ===== Config =====
RUN <- TRUE  # TRUE: process file; FALSE: run demo
ref_path <- "C:/Users/user/Downloads/summary-Schadendor-set.txt"
out_csv  <- paste0(sub("\\.txt$","", ref_path, ignore.case = TRUE),
"_journal_authors.csv")

## ===== Utils: robust read as UTF-8 =====
read_text_utf8 <- function(path) {
    if (!file.exists(path)) stop("File not found: ", path)
    ln <- readLines(path, encoding = "UTF-8", warn = FALSE)
    if (!length(ln)) stop("Empty file: ", path)
    iconv(ln, from = "", to = "UTF-8", sub = "byte")
}
```

Density(Dominance)

summary-Schadendor-set.txt

summary-Schadendor-set	2025/10/2 下午 01:31	文字文件	356 KB
summary-Schadendor-set_journal_aut...	2025/10/2 下午 01:35	Microsoft Excel ...	524 KB
summary-Schadendor-set_journal_su...	2025/10/2 下午 01:35	Microsoft Excel ...	1 KB

	A	B	C	D	E	F	G	
1	Journal	Author_1	Author_2	Author_3	Author_4	Author_5	Author_6	Auth
2	J Immunotl	Regan MM	Ascierto P/	Lipson EJ	Palaia J	Moshyk A	Selvan A	Lao
3	Eur J Canc	Ugurel S	Abu Rache	Gambichle	Mengoni M	T頓靖ing 1	Hassel JC	Resc
4	Eur J Canc	Leven AS	Peters T	Rajcsanyi I	Weichenth	Mohr P	Meier F	von
5	Future Onc	Schadendorf D						
6	medRxiv [1	J?μer N	Reuss DE	Sill M	Schrimpf D	Suwala AK	Sievers P	Bana
7	J Clin Onc	Wong MK	Milhem MI	Sacco JJ	Michels J	In GK	Mu蜴緻z (	Scha
8	Clin Cance	Lipson EJ	Dolfi S	Tang H	Tawbi HA	Medina-So	Castillo Gu	Rutk
9	Eur J Canc	DeTemple	Kaatz M	Stockfleth	Scheel C	Angela Y	Gutzmer R	Leite
10	Nat Comm	Kolb A	Kulis-Manc	Klein M	Stastny A	Haist M	Votteler V	Weic
11	Comput Str	Albahri M	Sauter D	Nensa F	Lodde G	Livingston	Schadendo	Kuku
12	Eur J Canc	Lipson EJ	Stephen Hc	Tawbi H	Schadendo	Ascierto P/	Matamala I	Gutie
13	J Clin Onc	Lodde GC	Hassel JC	von Wasiel	Meier F	Mohr P	K?νler K	Haus
14	J Transl Me	Borys K	Lodde G	Livingston	Weishaupt	R蝕雋er C	K頓雉nem	Helfe
15	J Transl Me	Borys K	Lodde G	Livingston	Weishaupt	R蝕雋er C	K頓雉nem	Helfe
16	PLoS One	von Bubno	Schmitt C	Goldinger	Schadendo	K?νler K(	Hafner C	Kran

Save it to country.csv

	A	B	C	D	E	F	G	H
1	Journal	Author_1	Author_2	Author_3	Author_4	Author_5	Author_6	Author_
2	J Immunotl	Regan MM	Ascierto P/	Lipson EJ	Palaia J	Moshyk A	Selvan A	Lao CD
3	Eur J Canc	Ugurel S	Abu Rache	Gambichle	Mengoni M	T頓靖ing 1	Hassel JC	Reschke
4	Eur J Canc	Leven AS	Peters T	Rajcsanyi I	Weichenth	Mohr P	Meier F	von Was
5	Future Onc	Schadendorf D						
6	medRxiv [1	J?μer N	Reuss DE	Sill M	Schrimpf D	Suwala AK	Sievers P	Banan R
7	J Clin Onc	Wong MK	Milhem MI	Sacco JJ	Michels J	In GK	Mu蜴緻z (	Schaden
8	Clin Cance	Lipson EJ	Dolfi S	Tang H	Tawbi HA	Medina-So	Castillo Gu	Rutkows
9	Eur J Canc	DeTemple	Kaatz M	Stockfleth	Scheel C	Angela Y	Gutzmer R	Leiter U
10	Nat Comm	Kolb A	Kulis-Manc	Klein M	Stastny A	Haist M	Votteler V	Weident
11	Comput Str	Albahri M	Sauter D	Nensa F	Lodde G	Livingston	Schadendo	Kukuk I
12	Eur J Canc	Lipson EJ	Stephen Hc	Tawbi H	Schadendo	Ascierto P/	Matamala I	Gutierre

https://raschonline.com/raschonline/cbpkano.asp?cbp=FLCA2allfiles
