# Supplementary material for: Mapping local cluster separation, global community structure, and leadership concentration among the 2025 top 2% dermatology scientists: Beyond rankings
Source: Medicine (Baltimore). 2026 Oct 9;105(41):e50851. doi: 10.1097/MD.0000000000050851 (PMC13657522; doi:10.1097/MD.0000000000050851)
Supplement: Supplementary file 2 [file medi-105-e50851-s004.pdf]

2.2 Interpretation of Silhouette and Modularity Metrics

2.2.1 Silhouette Score

The **silhouette score** [20,29], also known as the *cohesion–separation index*, measures two key concepts:

1. **Cohesion (a(i))** — the average distance between a data point *i* and all other points within the same cluster (C_I). Smaller values indicate higher within-cluster compactness:

$$a(i) = \frac{1}{|C_I|-1}\sum_{j\in C_I, j\neq i} d(i,j), \quad (1)$$

2. **Separation (b(i))** — the minimum average distance between a data point *i* and all points in any other cluster (*C_J*). Larger values indicate greater isolation from other clusters:

$$b(i) = \min_{J\neq I} \frac{1}{|C_I|-1}\sum_{j\in C_I, j\neq i} d(i,j), \quad (2)$$

The silhouette value *s(i)* combines these measures as:

$$s(i) = \begin{cases} \frac{b(i)-a(i)}{max[a(i),b(i)]}, & if \ |CI| > 1 \\ 0, & if \ |CI| = 1 \end{cases}, \quad (3)$$

The average silhouette of a cluster is:

$$\text{Overall SS} = \frac{1}{|C_I|}\sum_{i\in C_I} s(i), \quad (4)$$

Hence,

$$-1 \leq s(i) \leq 1 \qquad (5)$$

Interpretation:

- *s(i) ≈ 1*: point *i* is well matched to its cluster and clearly separated from others (high cluster quality).
- *s(i) ≈ 0*: unclear boundary; the point lies between two clusters.
- *s(i) < 0*: point may be misclassified into an inappropriate cluster

2.2.2 Modularity (Q)

Modularity (Q) quantifies how well a particular partition of a network deviates from a random (null) model, thereby assessing the quality of community division [31]. It measures the excess proportion of within-community edges compared with that expected in a random network preserving the degree distribution.

Interpretation:

- **Q > 0**: more within-community edges than expected — meaningful community structure.
- **Q ≈ 0**: partition is no better than random.
- **Q < 0**: fewer within-community edges than expected — partition conflicts with network structure.

Mathematically,

$$Q = \sum_{C} [\frac{l_C}{2m} - (\frac{d_C}{2m})^2], \qquad (6)$$

where

- ι_C= total weight of edges *within* community *C*,
- $d_C = \sum_{i \in C} k_i$= total degree (sum of node connections) in community

C,

- m= total weight (or number) of edges in the entire network.

The contribution of a single community *C* is:

$$Q_C = \frac{\iota_C}{2m} - (\frac{d_C}{2m})^2 \qquad (7)$$

For two independent communities with equal size and no interconnections, if

$$\frac{\iota_C}{2m} = \frac{d_C}{2m} = x,$$

then

$$Q_C = x(1 - x), \qquad (8)$$

which reaches its maximum when $x = 0.5$, i.e., when both communities are of equal size and fully independent, yielding $Q = 0.5$.

For *k* independent and equally sized communities, the maximum possible modularity is:

$$Q_{\max} = 1 - \frac{1}{k} \qquad (9)$$

This shows that $\mathbf{Q_{max}}$ increases with the number of balanced, independent clusters, representing the upper bound of achievable community quality.

2.2.3 Herfindahl–Hirschman Index and Simpson Diversity Index

In general form [33], Q in Equation (6) can be also expressed as Equation (10):

$$\boldsymbol{Q} = \sum_{i=1}^{k} (\boldsymbol{e}_{ii} - \boldsymbol{a}_i^2), (\mathbf{10})$$

where:

- e_{ii}= fraction of edges within community i,
- $a_i = \sum_j e_{ij}$= fraction of all edges that connect to nodes in community i,
- k= total number of communities (clusters).

The maximum theoretical modularity, $Q_{\max}$, occurs when:

- all edges lie *within* clusters (no inter-cluster edges),
- each cluster is completely isolated from others.

Under this ideal condition:

$$e_{ii} = \frac{1}{k}, a_i = \frac{1}{k}$$

Substitute these into the modularity formula:

$$Q = \sum_{i=1}^{k} \left(\frac{1}{k} - \left(\frac{1}{k}\right)^2\right) = k\left(\frac{1}{k} - \frac{1}{k^2}\right) = 1 - \frac{1}{k} = \mathrm{Q} = 1 - \sum_{i=1}^{k} (1/k)^2, (11)$$

Hence,

$$Q_{\max} = 1 - \frac{1}{k} \qquad (9)$$

- k= number of clusters (communities).
- $Q_{\max}$provides a theoretical ceiling for modularity given k.
- As $k$increases (more clusters), $Q_{\max}$approaches 1:

- $k = 2 \Rightarrow Q_{\max} = 0.5$
- $k = 5 \Rightarrow Q_{\max} = 0.8$
- $k \to \infty \Rightarrow Q_{\max} \to 1$

Because the achievable Q depends on k, analysts often normalize it:

$Q^* = \frac{Q}{Q_{\max}}$, (12)

so Q^*ranges from 0 (no structure) to 1 (maximally modular), allowing comparison between networks with different numbers of clusters.

Here, Equation (9) , $Q = 1 - \sum_{i=1}^{k} (1/k)^2$ in Eq. (9), resembles an extension of the Herfindahl–Hirschman Index (HHI) [34], expressed as:

$$HHI = \sum_{i=1}^{n} s_i^2 \qquad (12)$$

where s_i is the share (or proportion) of the i-th category (e.g., firm, author, or institution). The value ranges from 0 (complete dispersion) to 1 (complete concentration), as in measures of industrial concentration such as the steel industry [35].

Similarly, the Simpson's Diversity Index (D) [36] is defined as:

$$Simpson\ index = D = \sum_{i=1}^{n} p_i^2 \qquad (13)$$

Its complementary form, the Gini–Simpson Diversity Index (GS) [37], is given by:

$$1 - D = 1 - \sum_{i=1}^{n} p_i^2 \qquad (14)$$

where p_i represents the proportion of the i-th category. In bibliometrics, this index is commonly used to measure diversity among authors, institutions, or research topics, combining Simpson's D [36] into the GS metric [37]. Theoretical derivations and extensions of GS have also been discussed in ecological studies [38].

2.2.4 Interpretation of SS plot

The interpretation of SS plot is shown in Figure 1.

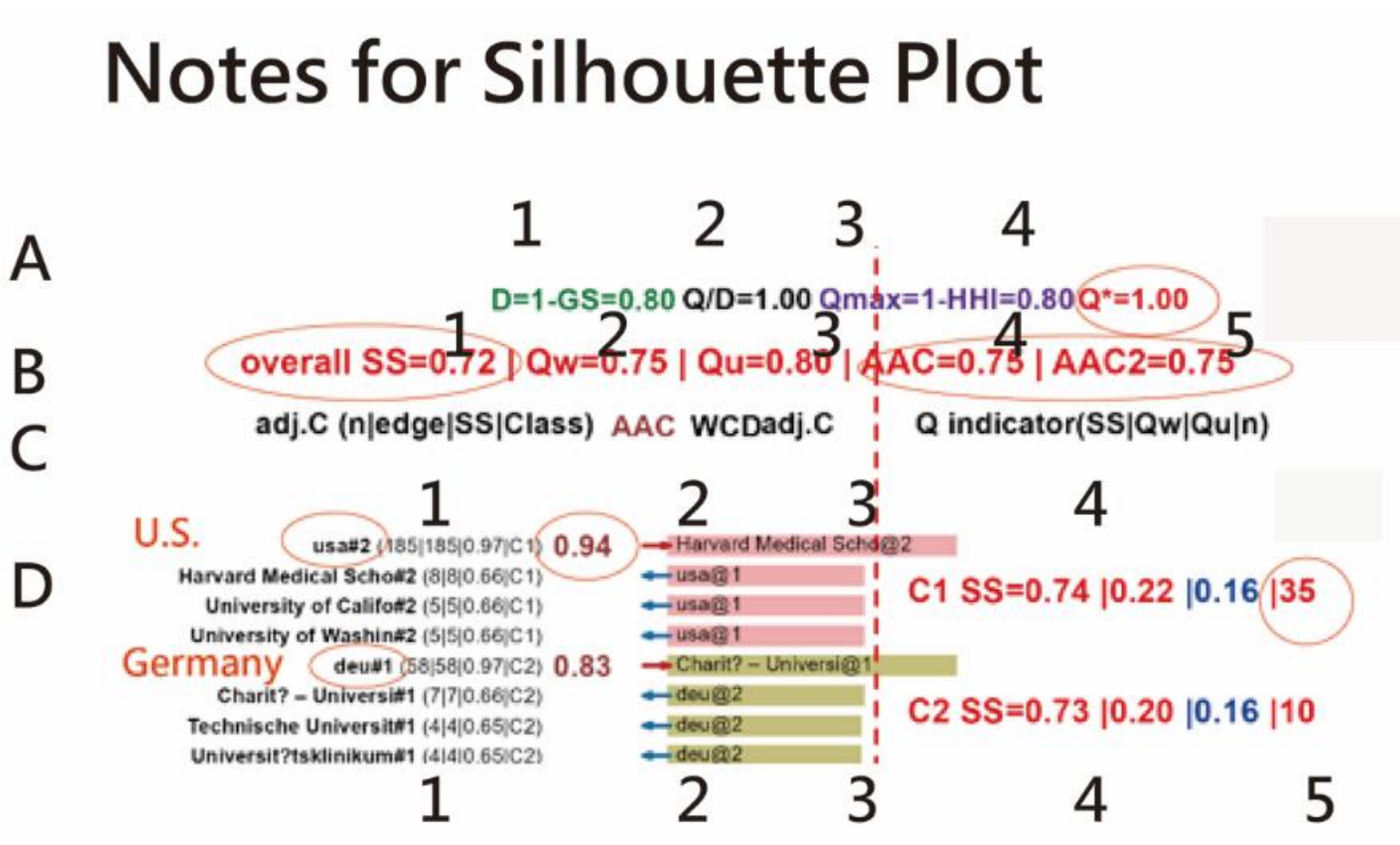

Figure 1 *Interpretation of the Silhouette (SS) plot (*The figure illustrates how SS values represent within-cluster cohesion and between-cluster separation, indicating cluster quality and classification accuracy)

In Figure 1A from 1 to 4, Gini–Simpson index=Gini_D= 1-D; Q/D=weighted Q/Gini_D; Qmax=max.Q=1-1/k, where, k is the number of non-link clusters; and Q*=Q/Qmax=weightedQ/max. possible Q

In Figure 1Bfrom 1 to 5, SS=weighted mean of all individual cluster SS;

weighted Q & unweighted Q; and Top 1 publication count AAC & collaboration AAC.

In Figure 1C from 1 to 4, Term & its nearest cluster # (count|collaboration|SS|class); Top 1 within cluster for collaboration AAC; WCD is the link counts, with the nearest neighbor @; Within cluster quality indicator(SS|Qw|Qu|n-count)

In Figure 1D from 1 to 5 as an example for contents, when WCD=1，meaning publications equal to collaborations; when SS & Q*>=0.5，meaning good clustering quality; and when SS & AAC>=0.7 for top 1, meaning dominance over next 2 terms.

2.2.5 Silhouette Plot

The silhouette plot reports the Absolute Advantage Coefficient (AAC) [30,39,40], as illustrated in Figures 1C and 1D. Using the Follower–Leading Clustering Algorithm (FLCA) [41], the silhouette plot visualizes the top 20 nodes.

The values in parentheses represent, in order: the number of publications, number of collaborations, silhouette score, and cluster label (e.g., C1, C2). A "#" symbol following a name denotes its most adjacent cluster. Subsequently, each cluster leader (the one with the highest collaboration frequency) is annotated with its AAC and Weighted Central Degree (WCD) [42]; the line thickness between nodes indicates collaboration strength (thicker lines = stronger relationships), and arrow direction

points from higher to lower collaboration volume. Neighboring nodes' cluster memberships are marked with "@" followed by their respective cluster IDs.

2.2.6 Reasons for Reporting Q*

Cluster quality is represented by the mean silhouette score (SS) and overall modularity (Q), as shown in Figure 1A. Because Q tends to increase with the number of clusters *k* (see Equation 9), it is necessary to report th**e normalized modularity,** defined as $Q^* = Q / Q_{max}$.

According to Equations (1)–(3), silhouette scores were calculated as shown in Figure 2. Distances were normalized by the reciprocal of co-occurrence frequency (i.e., higher co-occurrence → shorter distance; no co-occurrence = distance 1). Thus, each node's *a(i)*, *b(i)*, and *s(i)* values were computed; for example, the silhouette score for "term A" was 0.89.

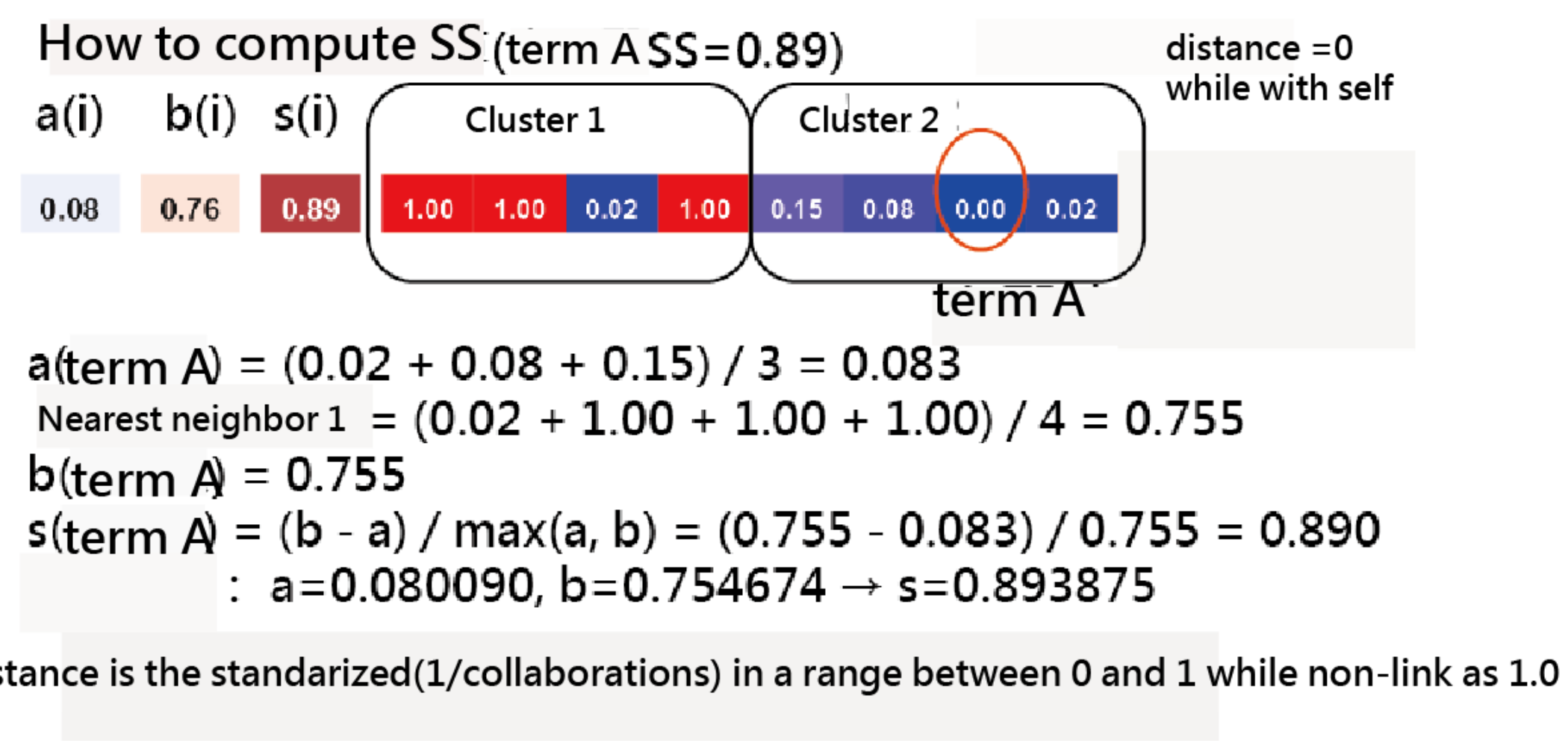

Figure 2 *Computation of the Silhouette Score (SS) — an example(*Demonstrates the calculation of cohesion a(i), separation b(i), and silhouette value s(i) for an example

term, showing normalization of co-occurrence distances and interpretation of resulting SS values)

References:

1. Ioannidis JPA, Baas J, Klavans R, Boyack KW. A standardized citation metrics author database annotated for scientific field. PLoS Biol. 2019 Aug 12;17(8):e3000384.

2. Yeung AWK, Tsoi JKH. A Critical Correlate of QS Subject Ranking and Stanford's Top 2% Scientists in Top Dental Schools. *Int Dent J*. 2025;75(5):100954.

3. Abdalla BA, Mustafa AM, Fattah FH, et al. Self-citation pattern among world's top 2 % of the scientists. *Heliyon*. 2025;11(3):e42471.

4. Ioannidis JPA, Boyack KW, Baas J. Updated science-wide author databases of standardized citation indicators. PLoS Biol. 2020 Oct 16;18(10):e3000918.

5. Wu AL, Chou W. Identifying China's distinctive academic fields among the top 2% of influential scientists: A bibliometric analysis using Rasch KIDMAP. Medicine (Baltimore). 2024 Jan 5;103(1):e36706.

6. Hirsch JE. An index to quantify an individual's scientific research output. Proc Natl Acad Sci U S A. 2005;102(46):16569-16572.

7. Walach H. Complementary Medicine Has Reached the Centre of Scientific Interest: Some Thoughts on Ioannidis' Citation Database. Complement Med Res.

2019;26(6):373-375.

8. Van Noorden R, Singh Chawla D. Hundreds of extreme self-citing scientists revealed in new database. Nature. 2019 Aug;572(7771):578-579.
9. Ioannidis JP, Klavans R, Boyack KW. Multiple Citation Indicators and Their Composite across Scientific Disciplines. PLoS Biol. 2016;14(7):e1002501.
10. Elsevier Data Repository. August 2024 data-update for "Updated science-wide author databases of standardized citation. 2025/9/20 at https://elsevier.digitalcommonsdata.com/datasets/btchxktzyw/7
11. Senior M, Fazel S. Measuring an individual researcher's impact: new directions and challenges. Evid Based Ment Health. 2020 May;23(2):47-51.
12. Slone RM. Coauthors' contributions to major papers published in the AJR: frequency of undeserved coauthorship. AJR Am J Roentgenol 1996;167:571–579.
13. Zbar A, Frank E. Significance of authorship position: an open-ended international assessment. Am J Med Sci 2011;341:106–109.
14. Jian D, Xiaoli T. Perceptions of author order versus contribution among researchers with different professional ranks and the potential of harmonic counts for encouraging ethical co-authorship practices. Scientometrics 2013;96:277–295.

15. Costas R, Bordons M. Do age and professional rank influence the order of authorship in scientific publications? Some evidence from a micro-level perspective. Scientometrics 2011;88:145–161.
16. Chien TW, Chou W. Visualizing leadership classifications in rectangular data using a basket model and co-word network analysis: a case study of U.S. HCAHPS survey results. BMC Med Res Methodol. 2025;25(1):195.
17. Kao WC, Chow JC, Chou W, Chen L. Discrepancy between countries most mentioned in earthquake-related articles and those most affected by major earthquakes since 1965: A bibliometric analysis. *Medicine (Baltimore)*. 2025;104(38):e43953.
18. Lim SW, Chou W, Chen L. SankeyNetwork: A clear and concise visualization tool for bibliometric data. *MethodsX*. 2025;14:103379.
19. Chien TW, Chou W. 10StepsinExcelforWoS: Streamlining bibliometric analysis in web of science using Microsoft excel. SoftwareX 2025; 32:102403. https://doi.org/10.1016/j.softx.2025.102403.
20. Chuang HY, Chou W. SilhouetteScoreinR: Beyond traditional network layouts by leveraging local cohesion and nearest neighbor separation. *MethodsX*. 2025;15:103622.

21. Blondel VD, Guillaume JL, Lambiotte R, Lefebvre E. Fast unfolding of communities in large networks. *J Stat Mech Theory Exp.* 2008(10):P10008. doi:10.1088/1742-5468/2008/10/P10008

22. Callon M, Courtial JP, Laville F. Co-word analysis as a tool for describing the network of interactions between basic and technological research. *Scientometrics.* 1991;22(1):155-205.

23. van Eck NJ, Waltman L. Software survey: VOSviewer, a computer program for bibliometric mapping. *Scientometrics.* 2010;84(2):523-538. doi:10.1007/s11192-009-0146-3

24. Newman MEJ. The structure of scientific collaboration networks. *Proc Natl Acad Sci U S A.* 2001;98(2):404-409. doi:10.1073/pnas.98.2.404

25. Perozzi B, Al-Rfou R, Skiena S. DeepWalk: Online learning of social representations. In: *Proceedings of the 20th ACM SIGKDD International Conference on Knowledge Discovery and Data Mining.* 2014:701-710. doi:10.1145/2623330.2623732

26. Grover A, Leskovec J. node2vec: Scalable feature learning for networks. In: *Proceedings of the 22nd ACM SIGKDD International Conference on Knowledge Discovery and Data Mining.* 2016:855-864. doi:10.1145/2939672.2939754

27. Kipf TN, Welling M. Semi-supervised classification with graph convolutional networks. In: *International Conference on Learning Representations (ICLR).* 2017.
28. Mikolov T, Chen K, Corrado G, Dean J. Efficient estimation of word representations in vector space. *arXiv.* 2013;1301.3781.
29. Rousseeuw PJ. Silhouettes: a graphical aid to the interpretation and validation of cluster analysis. J Comput Appl Math. 1987;20:53-65.
30. Li MJ, Lai FJ. Classifying research leadership in infantile hemangioma studies: A bibliometric analysis using Kano diagrams and absolute advantage coefficients. *Medicine (Baltimore)*. 2025;104(40):e44905.
31. Newman ME. Modularity and community structure in networks. Proc Natl Acad Sci U S A. 2006;103(23):8577-8582.
32. Chen C. CiteSpace II: Detecting and visualizing emerging trends and transient patterns in scientific literature. J Am Soc Inf Sci Technol 2006; 57 (3): 359-377.
33. Newman MEJ, Girvan M. Finding and evaluating community structure in networks. Phys Rev E. 2004;69(2):026113.
34. Herfindahl OC. Concentration in the U.S. Steel Industry [dissertation]. New York, NY: Columbia University; 1950.
35. Hirschman AO. The paternity of an index. Am Econ Rev. 1964;54(5):761–762.Herfindahl OC. Concentration in the U.S. Steel Industry. PhD Dissertation,

Columbia University, 1950.

36. Simpson EH. Measurement of diversity. Nature. 1949;163(4148):688.

37. Gini C. Variabilitàe mutabilità. Bologna, Italy: C. Cuppini; 1912.

38. Lande R. Statistics and partitioning of species diversity, and similarity among multiple communities. Oikos. 1996;76(1):5–13.

39. Yang DH, Chien TW, Yeh YT, et al. Using the absolute advantage coefficient (AAC) to measure the strength of damage hit by COVID-19 in India on a growth-share matrix. Eur J Med Res. 2021;26(1):61.

40. Yang TY, Chien TW, Lai FJ. Citation analysis of the 100 top-cited articles on the topic of hidradenitis suppurativa since 2013 using Sankey diagrams: Bibliometric analysis. Medicine (Baltimore). 2022;101(44):e31144.

41. Cheng YZ, Chien TW, Ho SY, Chou W. Visual impact beam plots: Analyzing research profiles and bibliometric metrics using the following-leading clustering algorithm (FLCA). Medicine (Baltimore). 2023;102(28):e34301.

42. Wu JW, Yan YH, Chien TW, Chou W. Trend and prediction of citations on the topic of neuromuscular junctions in 100 top-cited articles since 2001 using a temporal bar graph: A bibliometric analysis. Medicine (Baltimore). 2022;101(40):e30674.
